# Eviction from public housing in the United States

**DOI:** 10.1016/j.cities.2022.103749

**Published:** 2022-08

**Authors:** Ashley Gromis, James R. Hendrickson, Matthew Desmond

**Affiliations:** Sociology Department - Princeton University, Wallace Hall, Princeton University, Princeton, NJ 08544, USA

**Keywords:** Public housing, Eviction, Housing policy

## Abstract

Neither academic researchers nor the U.S. Department of Housing and Urban Development have studied evictions from public housing in national perspective. Combining federal registers of public housing authorities (PHAs) with individual-level records from >25 million eviction filings issued between 2006 and 2016, this is the first national-level study to estimate the prevalence and dynamics of eviction in public housing units. We find that the average PHA files roughly 40 evictions each year or 7.6 cases for every 100 public housing households. Public housing complexes were responsible for approximately 5.8 out of every 100 eviction filings in our sample, while only 3.5 in 100 renting households resided in public housing. Controlling for socioeconomic factors, we show that PHAs with a higher percentage of Black residents have significantly higher eviction filing rates. Eviction filing rates in PHAs are associated with those in the surrounding private rental market, indicating that PHAs do not function independently from the social contexts in which they are embedded. These findings reveal significant variation in eviction filing rates across local PHAs and highlight the need for clear policies on lease terminations and improved documentation of eviction actions in public housing at the federal and local levels.

## Highlights

• Court records and public housing data are linked to estimate eviction filings.
• Public housing authorities file a higher share of eviction cases than units managed.
• Public housing filing rates are associated with local non-public rental filing rates.
• Higher shares of Black residents are associated with higher filing rates.
• These associations are retained independent of tenant socioeconomic characteristics.

## 1 Introduction

For nearly a century, public housing has been a key feature of antipoverty policy in the United States. Throughout its history, government officials and housing advocates have affirmed that the intention of public housing is to shield severely disadvantaged households from the pressures of the private rental market that may otherwise result in threats to residential stability and displacement (see Hunt, 2005; McKenna & Hills, 1982; Office of U.S. Representative Ilhan Omar, 2019; Spence, 1993). Yet the U.S. Department of Housing and Urban Development (HUD), the agency responsible for overseeing these units, does not systematically collect or publish data on how many eviction cases are filed by public housing authorities (PHAs) in local courts or what share of public housing households are threatened with eviction each year.

All eviction filings are visible on a tenant's housing record, regardless of whether the tenant was ordered to vacate or removed from the premises, limiting access to future rental housing (Kleysteuber, 2006). A local study showed that roughly a third of tenants who receive an eviction notice leave before going to court (Desmond, 2016). The filings that do result in eviction deal a double blow to public housing tenants, resulting in the immediate loss of shelter as well as the loss of a substantial public benefit shown to reduce poverty and improve children's wellbeing (Andersson et al., 2016; Currie & Yelowitz, 2000). Understanding the extent to which public housing residents experience the threat of eviction is essential to informing policies aimed at preventing housing loss and promoting residential and community stability.

This study is the first to estimate the prevalence of eviction filings in public housing from a large sample of public housing authorities across several states. To estimate the prevalence of eviction filings in public housing, we combined individual-level court records of eviction cases with names and addresses of PHAs. We examined eviction case filings and public housing population characteristics for 1243 PHAs across 26 states. We used these data to determine (1) the prevalence of eviction filings in public housing; (2) the variation in eviction filing rates over time and across PHAs; and (3) whether socio-demographic characteristics associated with increased risk of eviction in the private rental market explain variation in PHA eviction filing rates. We found that the PHAs in our sample filed 328,845 eviction cases between 2006 and 2016, representing an average of 5.2% of residential eviction filings annually. There was greater variation in filing rates across PHAs than in private rentals located in the same areas, demonstrating substantial differences in rates at the PHA level. PHAs with higher shares of Black residents had higher filing rates net of several other socio-demographic conditions. This association remained even when comparing PHAs located in the same counties, indicating that public housing residents in predominantly Black complexes are disproportionately threatened with the loss of affordable housing.

## 2 Background

### 2.1 Public housing in the United States

For the first half of the 20th century, a patchwork of organizations at the federal and local levels coordinated the construction of subsidized housing units for low-income renters in the United States. In 1965, Congress established HUD, consolidating existing federal housing programs and funding local and state housing authorities (Erickson, 2009). Funding for public housing was significantly expanded by the Housing and Urban Development Act of 1968, resulting in a stock of >1.1 million units by 1974 (Erickson, 2009; McCarty, 2014). Construction of public housing units slowed in the 1970s, following reports of poor housing conditions and vacancies (Orlebeke, 2000). In 2022, HUD reported approximately 970,000 occupied public housing units in the United States, fewer than in the mid-1970s (McCarty, 2014; U.S. Department of Housing and Urban Development, 2022).

Previous research has shown higher residential stability among public housing tenants. The median length of residency in public housing was 4.7 years in 2000 (Lubell et al., 2003), compared to a median of two years among all renters in the United States (Mateyka & Marlay, 2010). A recent study drawing on a nationally representative study of children born in large cities between 1998 and 2000 found that children who lived in public housing at age nine reported lower rates of eviction during interviews at age 15 (Lundberg et al., 2020). Furthermore, surveys of public housing residents have consistently reported high levels of satisfaction with housing and neighborhood conditions (Lord & Rent, 1987; Varady & Preiser, 1998).

### 2.2 Eviction in public housing

Tenants who qualify for public housing are likely at heightened risk of falling behind on rent payments in the private housing market, the most common reason for eviction filings (Desmond, 2012). In public housing, rent payments are set at roughly 30% of monthly income (Schwartz & Wilson, 2008; Vale & Freemark, 2012). Despite this, a 20-year survey of a Hawaiian public housing board found that approximately three of every four eviction cases were filed for nonpayment (Monsma & Lempert, 1992), and a recent study of eviction cases in Philadelphia demonstrated that most cases filed against tenants in public housing buildings included nonpayment of rent as a reason for filing (Preston & Reina, 2021).

Over 1.8 million people live in public housing (Center for Budget and Policy Priorities, 2021), yet research on eviction from public housing is extremely limited. What little we know about the displacement prevalence and patterns in PHAs is based on a handful of local studies. These studies find that in some cities PHAs are important contributors to the overall number of eviction cases filed in local courts, even outpacing the share of the local rental market they manage. Between 2010 and 2019, the Philadelphia Housing Authority annually filed between 9 and 13% of eviction cases in the city, despite managing roughly 5% of the rental stock (Goldstein et al., 2019). While Preston and Reina (2021) found that residing in subsidized housing in Philadelphia was associated with lower risk of eviction filings when accounting for other building and neighborhood characteristics, public housing buildings had higher eviction filing risk compared with other types of subsidized properties. Data from the Atlanta metropolitan area showed that while eviction filing rates in senior, multi-family subsidized housing were lower than those in private market-rate apartments, there were no significant differences between the overall filing rate of subsidized housing and the private rental market (Harrison et al., 2020).

This study moves beyond local analyses by assembling a large sample of PHAs located across the United States to investigate the prevalence of eviction filings against public housing tenants. How many evictions are filed each year by PHAs, and do those filings represent a disproportionate share of cases? We also examine how eviction filing rates—the number of eviction filings divided by the occupied public housing units—vary over time and across PHAs.

Having uncovered considerable variation in PHA filing rates, we explore several possible explanations as to why some PHAs have significantly higher eviction filing rates than others. First, such variation could reflect a response to national economic trends, such as the Great Recession, when rates of material hardship increased across households (Gould-Werth & Burgard, 2012). Although public housing seeks to mitigate burdens imposed by housing costs, economic hardship may impact households' ability to pay even highly subsidized rent. Alternatively, public housing eviction filings could be more responsive to changes in PHA policies associated with violations of public housing leases. In the six months following the implementation of the “One Strike Policy” in 1996, which established a precedent for the swift removal of residents if any member of their household had engaged in criminal activity (Austin, 2002; Hellegers, 1999), PHAs denied almost double the number of prospective public housing tenants, owing to their criminal records (U.S. Department of Housing and Urban Development, 1997). The American Civil Liberties Union and Associated Press both released reports documenting significant increases in public housing evictions during this same period despite the lack of national data on eviction activity from HUD (Hoodridge & Strom, 2016; Waks, 2018). Lempert and Monsma (1988) documented increased numbers of cases commenced by subpoenas and eviction orders issued against public housing tenants in Oahu, Hawaii as the public housing board adopted more formalized administrative procedures and sought business and management experience in the appointment of new board members, suggesting that administrative changes can also affect eviction activity in PHAs. An increasing reliance on local courts to adjudicate lease violations could result in a higher prevalence of PHA eviction filings over time.

Spatial variation in filing rates may also reflect trends in eviction filings in the private rental market, which vary significantly across states. Many states in the Southeastern United States have significantly higher estimated filing rates, even after controlling for socio-demographic characteristics associated with eviction (Gromis et al., 2019). This likely reflects differences in state-level landlord tenant policy (Hatch, 2017), which determines the process for filing and prosecuting eviction cases (see also Merritt & Farnworth, 2020). Although public housing was developed as an alternative to the private rental market, geographic similarities between PHA and private rental eviction filing rates may suggest that PHAs are influenced by state or local eviction policies in their service areas.

Beyond larger temporal and spatial trends, previous research has identified many socio-demographic characteristics associated with an increased risk of eviction. Black renters, particularly Black women renters, have been consistently (and historically) shown to experience higher risk of eviction (Hartman & Robinson, 2003), at both the individual (Desmond, 2012; Desmond & Shollenberger, 2015) and neighborhood levels (Desmond, 2012; Medina et al., 2020; Merritt & Farnworth, 2020; Nelson et al., 2021). Family composition can also affect eviction risk, with female-headed households (Desmond, 2012) and households with children (Desmond et al., 2013) more likely to experience eviction. Increased economic hardship in female-headed households with children (Edin & Lein, 1997) could contribute to this elevated risk. These findings are also consistent with previous research demonstrating that pervasive discrimination based on race (Lempert & Monsma, 1994) and family composition (Allen, 1995; Desmond et al., 2013) are significant obstacles to tenants working to obtain or retain housing.

Economic conditions also affect eviction risk. Households experienced significant financial strain before receiving an eviction notice (Humphries et al., 2019). High-poverty neighborhoods in Milwaukee had an increased rate of eviction cases (Desmond, 2012), underscoring a link between financial precarity and housing instability. Variation in filing rates across PHAs (independent of larger temporal or spatial trends) could also reflect socio-demographic inequalities among public housing tenants, again suggesting that these units may not shield tenants from financial pressures or discriminatory practices operating in the private market.

Alternatively, it is also plausible that eviction filing rates vary across PHAs but are not associated with socio-demographic characteristics of the tenant population. PHAs operate independently, resulting in significant differences in housing policies at the local level, even within states (Lundgren et al., 2010). A shift away from federal administration over time has strengthened control of admission practices, management decisions, and operating procedures at the local, PHA level (Buckley & Schwartz, 2011). This includes policies that govern both the prioritization of applications beyond meeting the basic income requirements, including assigning preference to households with senior or disabled residents, very low incomes, or current residence in the PHA service area (Leopold, 2012; Martinez & Sard, 2000), and the expulsion of tenants (Renzetti, 2001), such as eviction policies related to criminal activity (Purtle et al., 2020).

Furthermore, limited expansion of low-income housing programs despite stagnant wages and rising rents has compromised the ability of poor families to locate stable, affordable housing, resulting in long waiting lists for subsidized units (Schwartz, 2014). Families spend an average of 26 months on waiting lists for federal rental assistance (Public and Affordable Housing Research Corporation, 2019). In 2012, 48.4% of PHA Housing Choice Voucher Programs waitlists were closed, the majority (65%) of which had been closed for more than a year (U.S. Department of Housing and Urban Development, 2012). Differential selection of tenants into public housing units, as well as the relative scarcity of these units, may also influence PHA filing rates.

## 3 Methodology

### 3.1 Methods overview

We combined four sources of administrative data on eviction records, PHAs, and population demographics to estimate the prevalence of eviction filings in public housing. We systematically identified public housing eviction filings by combining address and name information on PHAs maintained by HUD with tenant addresses and landlord names from a novel dataset of individual-level eviction records. We examined filings in public housing managed by PHAs, not in all housing subsidized by the federal government. Information on housing voucher and LIHTC units was not included in the HUD data, and we would not expect PHAs to be listed as plaintiffs on voucher or LIHTC filings. We restricted our analyses to data from 1243 PHAs (across 7821 PHA-years) with complete information on tenant socio-demographic characteristics located in counties where our eviction records represented a substantial majority (at least 86%) of court-reported case filings.

### 3.2 Eviction records

Our first source of data was a national database of individual-level records from >25 million eviction cases filed from 2006 to 2016 across all 50 states and the District of Columbia (DC).^3^ The records were provided by LexisNexis Risk Solutions and contained case-specific information, including the court in which the case was filed, court-assigned case number, dates associated with case actions, such as the case filing date, plaintiff (landlords) name(s), defendant (tenant) name(s) and addresses, and an indicator of whether the defendant represented an individual or business. Plaintiff names recorded the party who filed the case. In public housing cases, this is often the PHA name but could also be a property manager or other registered agent. This underscores the need to combine both name and address information to identify public housing cases.

Case filings were represented by the court identifier and case number. Many cases were represented by multiple individual-level records associated with different defendants or actions. We aggregated filings annually by the earliest date on a record associated with a case.^4^ The aggregates included all case filings, including multiple filings against the same household (i.e., serial filings).^5^ We assigned each case an address representing the property disputed in the eviction filing. Addresses were cleaned and geocoded using the 2016 Environmental Systems Research Institute (ESRI) USA Street Address Locator Files.^6^ We excluded any cases that had one or more commercial defendants as identified by the existing “business” indicator. We also removed cases that duplicated the same dates, plaintiff names, and tenant addresses across cases.

We validated the eviction filing data by comparing annual filings aggregated by county to aggregated filing counts from eviction case data received directly from the courts.^7^ There are many bureaucratic, legal, and practical barriers to collecting a complete set of eviction records (Desmond et al., 2018a, Desmond et al., 2018b; Hartman & Robinson, 2003). To ensure that we had adequate coverage of case data, we restricted our sample to only those counties in which our data showed a difference of ten or fewer filings or represented at least 86% of case filings reported in data obtained directly from the courts.^8^ In this way, we restricted our eviction records to unique, residential filings in geographic areas with a representative volume of case filings (*N* = 8,232,891 cases).

Some records also included case resolution information (e.g., a judgment restoring possession of the property to the landlord); however, this information was not available consistently for the counties with adequate filing coverage in our sample. For this reason, we measure case filings rather than case outcomes. Tenants may vacate a rental property at any point following the filing of an eviction case, rendering judgment information on these cases an incomplete indicator of both the threat and incidence of forced displacement. Filings appear in tenant housing screenings regardless of judgments issued on the case, making filings a barrier to securing stable and affordable housing (Kleysteuber, 2006). Additionally, our data did not include the reason for the filing (e.g., non-payment of rent, other lease violation), limiting our ability to examine why eviction cases are brought to court against public housing tenants.

### 3.3 Public housing authorities

Our second source of data was a listing of public housing building and unit addresses managed by PHAs from 2006 to 2016 (*N* = 3297 PHAs). We requested these data directly from HUD. We received one file including addresses of building entrances (many buildings contained several units) and a second file containing addresses for individual-level units. These data included full addresses, building opening and closing dates, and identifiers for the PHA that managed the properties and the housing project where the building was located.^9^ We combined the two files and geocoded the addresses using the 2016 ESRI USA Street Address Locator Files. Addresses that could not be geocoded at a precise street address were not used to identify PHA cases in the eviction records (6.6% of addresses). We linked the PHA identifiers to HUD's *Housing Authority Profiles* database to obtain the PHA names.^10^

We identified eviction cases filed by PHAs through four methods. First, we used regular expressions to identify common terms appearing in PHA names (e.g., “housing authority,” “housing agency,” “redevelopment commission”). Second, we matched the building entrance and unit addresses in the HUD files to tenant addresses in the eviction records. Third, we matched the PHA and housing development names in the HUD files to plaintiff names in the eviction records. Fourth, we included cases in which >50% of the eviction records with the same plaintiff name were identified as public housing cases based on address. Full details of the matching and assignment of cases to PHAs are available in Supplementary Information (SI), Section A.

We obtained information on the number of public housing units managed by PHAs and tenant population demographic characteristics from the HUD *Picture of Subsidized Housing* (our third data source). We downloaded available data at the PHA level for 2006 to 2016. These data included information on total number of units, percent of units occupied, race/ethnicity of tenants, presence of children in households, percent female-headed households, percent households with extremely low income (defined as having household income below 30% of local area median family income), age of the oldest householder, average time households spent on the waiting list, and size of the PHA. We calculated the total number of occupied households by multiplying the total number of units and the percent of units occupied.

To compare PHA filing rates and tenant population characteristics to those of the surrounding local area, we needed to determine the boundaries of the PHA service area. We did so by matching the PHA name to the city, county, and state where the associated building entrance or unit addresses were located (see Section A3 in SI). We excluded PHAs from the analysis that operated at the regional or state levels or had unknown service areas (*N* = 106). We assigned PHAs Federal Information Processing Standards (FIPS) codes from the 2010 Census consistent with their service area. For city-level PHAs, we assigned Census Designated Place (CDP) FIPS codes that corresponded to the city. For county-level PHAs, we assigned the FIPS codes for the corresponding county. We were unable to assign CDP FIPS codes for 47 city-level PHAs (1.9% of city-level PHAs).^11^ We excluded these PHAs from the analyses due to our inability to construct corresponding covariates from the eviction and Census data.

For the 2006–2016 period, the 3144 city- or county-level PHAs could each contribute a possible 11 years of observations (*N* = 34,584 PHA-years). We restricted our analytic sample to those PHA-years with adequate coverage in both the eviction and *Picture of Subsidized Housing* data. For the eviction data, we included only PHAs in counties with validated filing coverage, as described above (*N* = 1754 PHAs; 10,462 PHA-years).^12^ Not all PHAs consistently reported data to *Picture of Subsidized Housing*. We excluded PHA-years that were missing data or contained incomplete data (*N* = 1398 PHA-years). We required that at least 80% of occupied PHA units contributed to the reported data to ensure we had a representative picture of the tenant population and excluded PHA-years that did not meet this threshold (*N* = 31 PHA-years).^13^ Finally, to maintain consistency over time and across space in the PHA sample, we imposed the additional restriction that PHAs met the above requirements for at least two consecutive years and that there were at least five PHA observations in each state-year. Our analytic sample included 7821 PHA-year observations across 1243 PHAs in 26 states (SI Fig. D1).

### 3.4 Socio-demographic characteristics of PHA service area

Our fourth source of data was socio-demographic characteristics from the U.S. Census and American Community Survey (ACS). We used these measures primarily to control for the socio-demographic composition of the PHA service area. We used data from the 2000 and 2010 Censuses and 2016 ESRI Business Analyst to estimate the number of renter households in each CDP or county. We downloaded data at the Census block group level and linearly interpolated between the three time points (2000, 2010, and 2016) before aggregating the block groups into CDPs and counties. We downloaded additional covariates directly from the Census and ACS, including population by race/ethnicity, percent female-headed households, percent households with children under age 18, poverty rate, unemployment rate, median property value, and percent renter occupied households. SI Table C1 shows how ACS and Census waves were assigned to the years in our analysis. All observations in our analytic sample had complete coverage on the Census and ACS covariates.

### 3.5 Eviction filing rates

We calculated eviction filing rates for PHAs and private market rental housing in the PHA service area (city or county). To calculate PHA filing rates, we divided the number of PHA case filings by its total number of occupied public housing units. To calculate comparable private rental market rates, we divided the number of non-public housing cases by the number of non-public renter households in the service area. We calculated the number of non-public renter households by subtracting the number of occupied public housing units from the total number of occupied renter households. We calculated all filing rates annually.

### 3.6 Regression analyses

We estimated the effects of socio-demographic characteristics of both PHA tenant population and the full population in the PHA service area on the PHA filing rate. We used a longitudinal linear regression model with random effects at the PHA level. This allowed us to examine how PHA and tenant population characteristics were associated with filing rates across PHAs while accounting for repeated measures from the same PHAs over time. Our outcome variable was the logged PHA filing rate. We took the natural log of the filing rate to adjust for skew (many PHAs file very few eviction cases while others file more eviction cases annually than they have occupied units).^14^ We included fixed effects for both year and state. Year fixed effects helped absorb time trends in eviction filings that may be correlated with other covariates in the models. State fixed effects accounted for time-invariant state-level differences previously observed in filing rates (Gromis et al., 2019). All models specified robust standard errors clustered by PHA.

We included socio-demographic characteristics of the PHA tenant population shown to be associated with eviction in previous studies, including the share of Black tenants, share of Hispanic tenants, percent of households with children, percent female-headed households, household income, and percent of households with the oldest householder at least 65 years of age (Desmond, 2012; Desmond et al., 2013; Harrison et al., 2020; Medina et al., 2020; Merritt & Farnworth, 2020; Nelson et al., 2021). We also included the average number of months spent on the waiting list. Increased demand (resulting in longer wait times for units) could result in PHAs being more likely to file eviction cases against tenants behind on rent or in violation of lease terms. We controlled for the size of the PHA, measured in five ordinal categories: 1–99 units, 100–299 units, 300–499 units, 500–999 units, and 1000 or more units. Larger PHAs may have more resources to file and prosecute eviction cases, even after adjusting the number of filings by the number of occupied households. For this reason, we might expect larger PHAs to have higher filing rates than small PHAs, other factors equal.

The models also included socio-demographic characteristics of the surrounding PHA service area, including share of Black and Hispanic tenants, percent female-headed households, poverty rate, percent employment, percent renting households, percent households with children, and property values. We included this information for two reasons. First, neighborhood-level variables acted as controls to ensure that any associations between PHA tenant characteristics and the eviction filing rate reflected the characteristics of the public housing population, not the those of the larger population in the PHA service area. Second, it is possible that conditions in the surrounding area affected how often a PHA files eviction cases. Studies have shown that a neighborhood's racial composition is associated with nuisance complaints (Desmond & Valdez, 2013) and crime enforcement (Weitzer, 2000), conditions that could be associated with evictions in public housing.

Finally, we controlled for the eviction filing rate in non-public housing in the PHA service area. Examining the association between the non-public and PHA filing rates, net of socio-demographic characteristics of the tenant and larger population, helped us investigate how closely trends public housing eviction filings approximate those in the private rental market. A positive association between these two rates would suggest that PHA eviction behavior may be influenced by the same local and state policies that shape landlord behavior in the private rental market. Alternatively, a lack of association (or an inverse association) would suggest that public housing lease terminations operate independently of the forces likely to govern private landlord eviction behavior, such as the housing market and landlord-tenant policy. Descriptive statistics for all variables included in the regression models are shown in Table 1. Comparative statistics for PHA-years that were not included in the analytic sample are shown in SI Table C2. Overall, PHA-years excluded from analytic sample have a slightly higher mean number of occupied units annually but otherwise share very similar characteristics to PHA-years in analytic sample.

**Table 1:** Descriptive statistics for PHA-years in analytic sample, 2006–2016.

Variable	N	Mean	Std. Dev.	Median	Min	Max
Public Housing Authority						
Total units	7821	311.68	783.43	100.00	12.00	10,490.00
Occupied units	7821	289.51	713.56	97.00	10.95	10,079.30
Eviction filings	7821	42.05	164.72	1.00	0.00	3311.00
Eviction filing rate	7821	7.64	15.24	1.67	0.00	183.67
% Black tenants	7821	33.14	36.18	15.00	0.00	100.00
% Hispanic tenants	7821	8.17	17.56	1.00	0.00	100.00
% Households with children	7821	37.11	22.39	38.00	0.00	100.00
% Female-headed households	7821	74.21	11.03	75.00	19.00	100.00
% Households extremely low income	7821	63.17	16.47	65.00	0.00	100.00
% Householders aged 65+	7821	34.66	21.43	29.00	0.00	100.00
Months on waiting list (mean)	7821	10.70	13.92	6.00	0.00	255.00
Size						
1–99 units	3081	0.00			0.00	1.00
100–299 units	1941	0.00			0.00	1.00
300–499 units	729	0.00			0.00	1.00
500–999 units	817	0.00			0.00	1.00
1000+ units	1253	0.00			0.00	1.00
Service Area						
% Black population	7821	14.69	18.75	5.77	0.00	95.48
% Hispanic population	7821	9.89	15.09	3.93	0.00	99.52
% Female-headed households	7821	13.91	5.78	12.86	1.83	43.86
% Families in poverty	7821	14.68	7.29	13.68	0.00	49.58
% Unemployed	7821	8.44	4.04	7.85	0.00	32.83
% Renting households	7821	35.28	11.36	33.99	6.77	81.61
% Households with children	7821	31.07	5.84	30.86	9.19	69.76
Property value (median, in $1000s)	7821	126.01	78.47	105.40	28.00	997.01
Eviction filing rate	7821	5.57	7.22	3.12	0.00	81.76

Note: All measures calculated annually and averaged across years in sample. Eviction filing rates (ratio of case filings to renting households) can occur in excess of 100% due to repeated filings against the same households or turnover in units.

To better understand variation in PHA eviction filing rates, we also estimated a secondary regression model in which we more explicitly compared PHAs located within the same county. A county may have multiple PHAs for two primary reasons: (1) PHAs most commonly operate at the city level and many counties contain multiple cities and (2) some counties have both city-level and county-level PHAs. We included cities in which 95% or more of their area was contained within a single county. This model only included instances in which we had at least two PHA observations in the same county-year (*N* = 4393 PHA-years across 705 PHAs).^15^ We relied on the same set of covariates described above and included fixed effects for year and county (rather than the state) level. This should have controlled for time-invariant local jurisdiction characteristics and other within-county-year conditions that could not be measured in the model and helped isolate effects of PHA tenant population characteristics.

## 4 Results

**Fig. 1:**
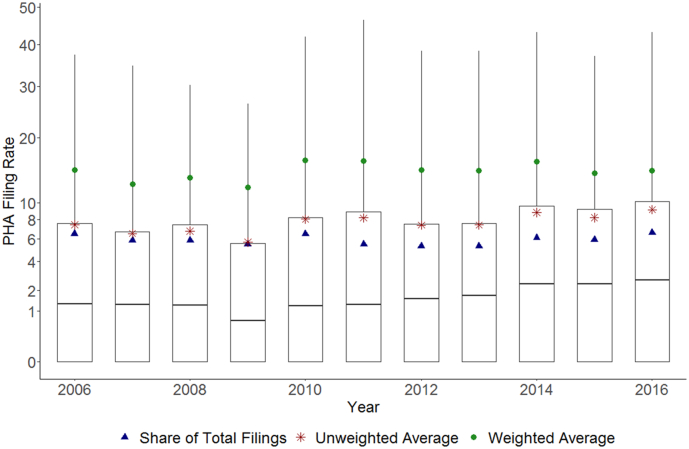
Distribution of PHA filing rates with summary statistics for share of total filings and unweighted and weighted average filing rates, 2006–2016. Note: Box plots show 5th through 95th percentiles of PHA filing rates annually. Lower bound of box marks 25th percentile, middle line the median (50th percentile), and upper bound of box the 75th percentile. Summary statistics—share of total eviction filings attributable to PHAs, unweighted mean PHA filing rate, and weighted PHA mean filing rate—shown by points. Y-axis plotted on square root scale due to right skew present in distribution of filing rates.

The 1243 PHAs in the analytic sample filed an average of 29,895 eviction cases annually (SI Fig. D2). This amounted to 42 filings per year for the average PHA, or approximately 7.6 cases per every 100 public housing renting households (Table 1). The distribution of average (unweighted) filing rates has remained relatively stable over time (Fig. 1). The longitudinal sample of PHAs was unbalanced (i.e., the same PHAs are not included across all years), which makes direct comparisons across years difficult; however, state-level average filing rates across PHAs consistently captured in the sample showed largely consistent levels of filings across years (SI Fig. D3). Weighing the average filing rate by number of occupied housing units resulted in a higher average filing rate in all years. Weighted average filing rates are nearly twice that of unweighted rates, demonstrating that larger PHAs tend to have higher filing rates (average filing rates by PHA size are shown in SI Fig. D4).^16^

The share of total filings attributable to public housing also remained stable over time (range: 5.3% to 6.6%). Annually, PHAs in our sample were responsible for 5.8 out of every 100 eviction filings but managed only 3.5 of every 100 rental units, on average. The average PHA filing rate patterns roughly track the filing rates in non-PHA (privately-managed) rental units across years (SI Fig. D6). While selection into public housing limits the comparisons that can be made to filing rates in the private rental market, these findings reveal that PHAs file a substantial number of eviction cases each year and that they are responsible for a disproportionate share of eviction filings.

**Fig. 2:**
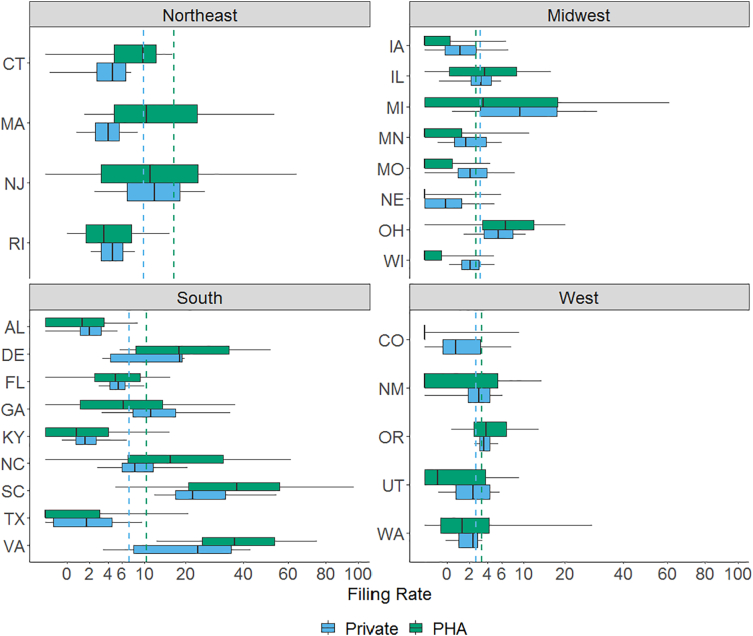
The distribution of PHA and private rental unit filing rates, by state. Note: Box plots show 5th through 95th percentiles of PHA filing rates annually. Lower bound of box marks 25th percentile, middle line the median (50th percentile), and upper bound of box the 75th percentile. States are grouped by Census region. Dotted lines show average filing rates for private (blue) and PHA (green) units in each region. Y-axis plotted on square root scale due to right skew in distribution of filing rates. (For interpretation of the references to colour in this figure legend, the reader is referred to the web version of this article.)

PHA filing rates vary significantly, both within and across states (Fig. 2). Many PHAs had very few (or no) annual eviction filings, while others routinely had filing rates in excess of 10% of occupied units (i.e., at least one filing for every ten households). PHA filing rates showed greater variation than private market filing rates in almost all states. Although smaller numbers of public housing units relative to private market rentals create the opportunity for more extreme rates, high PHA filing rates are not driven by the smallest denominators (SI Fig. D4). Furthermore, while selection into public housing may concentrate households vulnerable to eviction filings, the lack of uniformly high filing rates across PHAs suggests that there is not a straightforward relationship between aggregate socio-economic risk and likelihood of eviction filings.

Average PHA filing rates were highest in the Northeastern and Southern regions, although all states contained PHAs with filing rates above and below the annual PHA average (7.6%). Even within regions, median filing rates in PHA and private rental units tended to correspond within states. The highest median filing rates (>20%) for both PHA and private rentals are found in South Carolina and Virginia. The variation in PHA filing rates within and across states suggests that threats to displacement from public housing do not operate independently from local economic and policy conditions.

### 4.1 Within-state PHA-level analyses

**Fig. 3:**
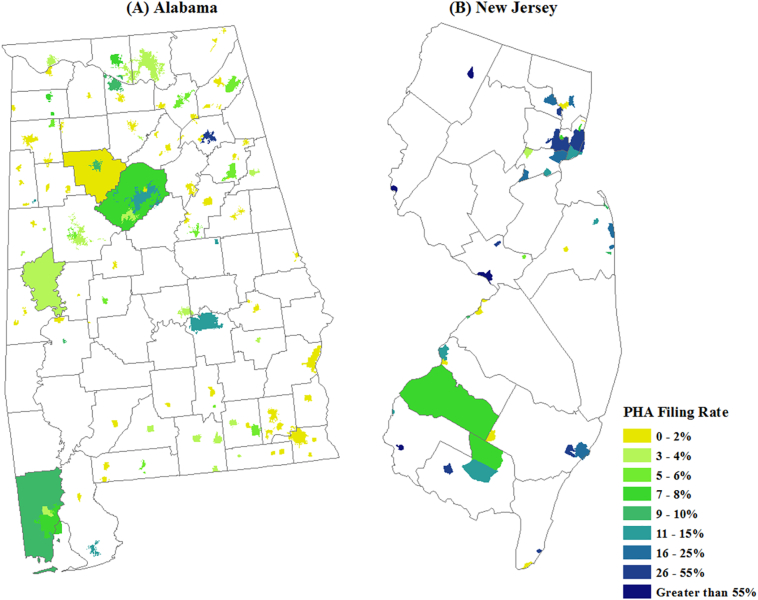
PHA filing rates in (A) Alabama and (B) New Jersey, 2016.

To illustrate different patterns of PHA filing rates within states, the rates for PHAs in Alabama and New Jersey are shown in Fig. 3. Although both states contain PHAs with below average filing rates in 2016, these lower rates are more common across places and counties (both rural and urban) in Alabama. New Jersey, which has the highest median PHA filing rate in the Northeast Region (Fig. 2), showed the opposite pattern—filing rates were consistently high across many PHAs, regardless of location in the state.

To examine variation in filing rates across PHAs within states, we estimated a longitudinal model with random effects at the PHA level. This model allowed us to examine associations between characteristics of the PHA tenant population, larger population in the PHA service area, and the PHA filing rate, while accounting for repeated observations from the same PHAs. The model included state and year fixed effects to help account for time-invariant interstate differences in filing rates as well as any baseline differences in filing rates over time. The results from this model are presented in Table 2.

**Table 2:** Associations between PHA and service area characteristics and PHA filing rate, 2006–2016.

Variable	Coeff.	SE	Sig.
Public Housing Authority			
% Black tenants (in 10%)	0.095	0.032	0.003⁎⁎
% Hispanic tenants (in 10%)	−0.086	0.047	0.065
% Households with children (in 10%)	0.089	0.042	0.037⁎
% Female-headed households (in 10%)	−0.032	0.047	0.503
% Households extremely low income (in 10%)	0.069	0.030	0.023⁎
% Householders aged 65+ (in 10%)	−0.193	0.041	0.000⁎⁎⁎
Months on waiting list	−0.004	0.003	0.083
PHA size			
1–99 units	−1.852	0.178	0.000⁎⁎⁎
100–299 units	−0.661	0.166	0.000⁎⁎⁎
300–499 units	(reference)		
500–999 units	0.134	0.170	0.432
1000+ units	0.569	0.175	0.001⁎⁎
Service Area			
% Black population (in 10%)	−0.086	0.061	0.157
% Hispanic population (in 10%)	−0.046	0.065	0.472
% Female-headed households	0.014	0.014	0.298
% Families in poverty	0.002	0.009	0.837
% Unemployed	0.004	0.013	0.748
% Renting households	0.009	0.006	0.149
% Households with children	0.009	0.009	0.324
Property value (in $1000s)	0.001	0.001	0.235
Eviction filing rate (logged)	0.098	0.024	0.000⁎⁎⁎
Constant	−1.431	0.554	0.010⁎
R^2^ = 0.570 (overall)			
*N* = 7821 PHA-years (1243 PHAs)			

Estimates generated in longitudinal linear regression model with random effects at the PHA level. Fixed effects for states and years were included in the model but not shown in the table due to space considerations.

⁎⁎⁎ *p* < 0.001.

⁎⁎ *p* < 0.01.

⁎ *p* < 0.05.

Several socio-demographic characteristics of the PHA tenant population were associated with PHA filing rates. First, larger shares of Black residents in PHA units were associated with higher filing rates. For each 10% increase in the share of Black tenants, PHA filing rates were expected to increase by 10.0%, holding constant other socio-demographic characteristics of the tenant population and service area. Larger shares of households with children were also associated with higher filing rates. A 10% increase in households with children was associated with a 9.3% increase in PHA eviction filing rates, holding constant other socio-demographic characteristics of the tenant population and service area. For each additional 10% of the tenant population that is extremely low income (<30% of the local area median family income), we would expect a 7.1% increase in PHA filing rate, net other socio-demographic characteristics of the tenant population and service area. Finally, PHAs with higher shares of householders at least 65 years of age were also expected to have lower filing rates: each additional 10% of tenant households aged 65 or older was associated with an expected decrease of 17.6% in PHA filing rate. Percent Hispanic population, female-headed households, and months spent on the waitlist were not significantly associated with PHA filing rates. These findings are consistent with previous research showing positive associations between larger shares of African American population, households with children, and poverty and eviction risk in the private rental market (Desmond, 2012; Desmond et al., 2013) and a negative association between senior, subsidized housing and eviction filing rates (Harrison et al., 2020).

PHA size was also associated with filing rates. Larger PHAs filed eviction cases at higher rates than smaller PHAs, even after adjusting for the number of occupied units. This may stem from increased resources to file cases or to employ managers who rely on civil courts as arbitrators of disputes. This could also reflect the inability or unwillingness of larger PHAs to handle nonpayment of rent or potential lease violations on a tenant-by-tenant basis.

To further assess how tenant population composition may have affected eviction filing rates, we completed a supplemental analysis using a survey of PHA public housing waitlist preferences in 2012 (see SI Section B for details of these data). PHAs have discretion to prioritize waitlist placement for specific applicants (e.g., senior/disabled tenants, those experiencing homelessness) which could affect selectivity of the tenant population, vulnerability to missed rent payments, and overall risk of eviction filings. We were able to match the cross-sectional waitlist data to 1002 PHAs in our sample. We then fit the same regression models as presented in Table 2, with the additional indicators of whether PHAs reported preference placements on the waitlists. We found no statistically significant associations between waitlist preferences for extremely disadvantaged groups and filing rates (SI Table B1), indicating that variation in PHA filing rates is likely not a function of selection of the most socio-economically disadvantaged households into some units.

None of the characteristics of the larger population in the PHA service area showed significant associations with PHA filing rates, including traditional indicators of neighborhood socio-economic disadvantage. There was a strong positive relationship between the eviction filing rate in the private rental housing market of the PHA service area and the PHA filing rate. Public and private eviction filing rates seem to move in lockstep, such that for every 1% increase in the private rental filing rate, the PHA filing rate is expected to increase by 1.0%, net of characteristics of the public housing population, PHA, and larger population in the service area. PHAs do not appear to be operating independently of the policy context and housing conditions that shape eviction dynamics in local private rental markets.

### 4.2 Within-county PHA-level analyses

**Fig. 4:**
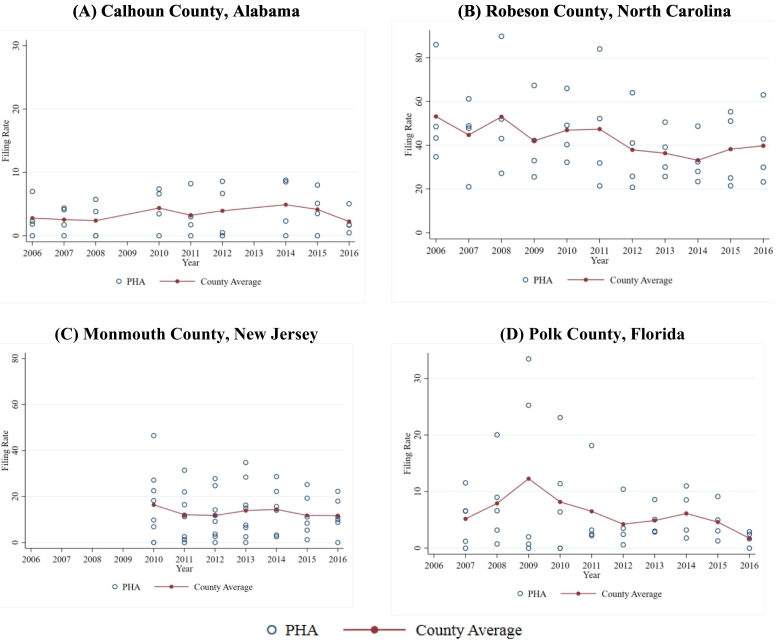
Within-county variation in PHA filing rates, 2006–2016. Note: Circles represent individual PHA filing rates. Connected dots show the average PHA filing rate in the county. (A) Calhoun County, Alabama (B) Robeson County, North Carolina (C) Monmouth County, New Jersey (D) Polk County, Florida.

The association between eviction filing rates in private rentals and public housing suggests that unmeasured county-level characteristics may be exerting influence over the frequency of eviction case filings by PHAs. We further isolated PHA-level variation from between-county variation by comparing filing rates in PHAs located in the same county (Fig. 4). In any given year, PHAs in the same county exhibit substantially different eviction filing rates. Some counties showed consistently low (Panel A) or high (Panel B) PHA filing rates; however, other counties exhibited substantial, persistent differences in filing rates across PHAs (Panel C) or substantial, fluctuating differences in filing rates across PHAs over time (Panel D). These results indicate that while PHAs do not appear to operate independently of local housing or policy conditions, unmeasured county-level differences are not sufficient to explain variation in filing rates across PHAs.

To investigate how PHA-level characteristics affect filing rates within the same local context, we conducted a secondary analysis with a restricted sample that included only county-years with at least two PHA observations. We fit the same regression model described above, but with county (rather than state) fixed effects (Table 3). None of the socio-demographic characteristics in the PHA service area were significantly associated with PHA filing rates. The filing rate among private rentals in the PHA service area was still positively associated with PHA filing rates, although only marginally so.

**Table 3:** Associations between PHA and service area characteristics and PHA filing rate for multiple PHA observations within the same county, 2006–2016.

Variable	Coeff.	SE	Sig.
Public Housing Authority			
% Black tenants (in 10%)	0.168	0.050	0.001⁎⁎
% Hispanic tenants (in 10%)	0.016	0.065	0.804
% Households with children (in 10%)	0.082	0.067	0.222
% Female-headed households (in 10%)	0.018	0.069	0.798
% Households extremely low income (in 10%)	0.027	0.044	0.533
% Householders aged 65+ (in 10%)	−0.143	0.061	0.019⁎
Months on waiting list	−0.007	0.003	0.019⁎
PHA size			
1–99 units	−1.920	0.263	0.000⁎⁎⁎
100–299 units	−0.773	0.234	0.001⁎⁎
300–499 units	(reference)		
500–999 units	−0.032	0.256	0.900
1000+ units	0.442	0.265	0.095
Service Area			
% Black population (in 10%)	−0.107	0.095	0.262
% Hispanic population (in 10%)	−0.018	0.105	0.866
% Female-headed households	0.003	0.020	0.879
% Families in poverty	0.016	0.014	0.230
% Unemployed	−0.001	0.020	0.967
% Renting households	−0.002	0.010	0.878
% Households with children	−0.002	0.016	0.924
Property value (in $1000s)	0.000	0.001	0.779
Eviction filing rate (logged)	0.074	0.036	0.043⁎
Constant	−2.644	1.673	0.114
R^2^ = 0.634 (overall)			
N = 4393 PHA-years (705 PHAs)			

Estimates generated in longitudinal linear regression model with random effects at the PHA level. Fixed effects for counties and years were included in the model but not shown in the table due to space considerations.

⁎⁎⁎ p < 0.001.

⁎⁎ p < 0.01.

⁎ p < 0.05.

Table 3 shows that two characteristics of the PHA tenant population—the share of Black residents and share of senior (65 or older) householders—maintained significant associations with eviction filing rates. In this model, for every additional 10% increase in share of Black residents, the PHA filing rate was expected to increase by 18.3%, holding constant other socio-demographic characteristics of the tenant population and service area. This demonstrates that the relationship between higher shares of Black population and higher PHA eviction filing rates is unlikely to be explained by unmeasured county-level socio-demographic or policy characteristics. Likewise, a 10% increase in the share of householders aged 65 or older was associated with a predicted 13.3% decrease in PHA filing rates, net of other factors. The associations for share of households with children and extremely low income resembled the direction of the effect in the full model but were imprecisely estimated in this sample-restricted model.^17^

In these models, PHA size and time on the waitlist before receiving a housing unit also showed significant relationships with PHA filing rates. Other factors equal, PHAs with 300 or more units are expected to file more eviction cases than smaller PHAs. Contrary to our expectations, increased waitlist times were associated with lower filing rates across PHAs within the same county. This finding could reflect a reinforcing relationship: if a PHA was evicting a higher number of tenants, tenant turnover may increase, reducing the average amount of time households spent on the waitlist. This inverse relationship between PHA waitlists and filing rates could also result from increased selectively of tenant populations in PHAs with longer waitlists owing to admission criteria or increased tenant efforts to retain their public housing unit, given the scarcity of the resource.

The limited availability of data on PHA policies and operating procedures makes it impossible to fully evaluate how characteristics of the PHA itself influenced how often tenants receive eviction filings. We were able to match the limited waitlist prioritization survey data to 358 PHAs in our within-county sample. Having done so, we continued to find no significant differences in filing rates based on prioritizing the most economically-disadvantaged households (second column SI Table B1).

There are also many political factors, including how members of housing boards are appointed, accountability of housing boards to residents, community representation on these boards, and relative autonomy (or lack of) between PHA leadership and local governments that may influence eviction filing activity. Important early work by Monsma and Lempert documented fluctuations in eviction filing rates following changes in the structure and composition of a public housing eviction board in Hawaii (1988) and cultural discrimination against Samoan tenants in eviction hearings due to lack of Samoan representation on Hawaiian housing boards (1994). More recent work has also established an association between Black municipal leadership and the transformation of public housing, including the demolition of public housing units (Owens et al., 2021), again suggesting an important connection between local politics, representation, and the operation of public housing. Limited availability of data—administrative, survey, or otherwise—on the governance and administration of public housing prevents us from incorporating these characteristics into the current study. Collection and examination of these measures represent an important avenue for future research on the frequency of and disparities in eviction filings (and displacement due to eviction) across PHAs.

## 5 Conclusions

### 5.1 Discussion

This study has provided the first estimates of the annual prevalence of eviction filings in public housing in the United States. Collectively, the 1243 PHAs in our sample managed 3.5 in every 100 of the renter households but were responsible for 5.8 in 100 eviction filings. Selection into public housing concentrates tenants that may be vulnerable to eviction; however, if the aim of public housing is to promote residential stability, an aggregate filing rate that outpaces the share of tenants residing in these units warrants investigation. Our results show that tens of thousands of households are threatened with eviction from public housing each year.

PHA filing rates were relatively consistent over time and did not deviate significantly from patterns in private rental filing rates. We demonstrated a correspondence between differences in PHA and private rental filing rates across states. The persistent significant association between PHA filing rates and those among private rental units in the surrounding service area suggests that the former are responsive to local housing and policy conditions. This indicates a general connection between eviction filing dynamics in public housing and the surrounding private rental market. Consistent with this finding, we did not find consistent associations between socio-economic characteristics of the tenant population and PHA filing rates.

Disproportionate representation in public housing eviction records may place significant burden on households already likely to face discrimination in the private rental market. An eviction from public housing can be used to prohibit tenants from benefitting from subsidized housing in the future, denying families much-needed rental assistance (Curtis et al., 2013). Our findings show that tenants in PHAs with higher shares of Black residents are disproportionally exposed to eviction risk and its consequences, further limiting their ability to secure safe and affordable housing.^18^ It is possible that tenants in these PHAs committed more eviction-warranting action, such as nonpayment. However, the association between share of Black public housing residents and higher PHA filing rates persisted even after controlling for the share of tenants with extremely low incomes and socio-demographic characteristics of the PHA service area, as well as after comparing PHAs within the same counties. This indicates that traditional explanations of eviction risk focused on economic disadvantage are likely not responsible for this finding. A more likely explanation has to do with an acute reliance on eviction proceedings in predominantly-Black PHAs that parallels punitive strategies adopted by other government institutions that disproportionately supervise low-income Black populations, including criminal-legal agencies (Kohler-Hausmann, 2018; Western et al., 2021) and welfare offices (Schram et al., 2008; Watkins-Hayes, 2009). The discrepancy in the rate of eviction filings in PHAs with a large and small share of Black residents may be driven by putatively race-neutral policies or procedures that generate racial disparities on a large scale. For example, PHAs with higher shares of Black residents may be more likely to contract private management companies, increasing the visibility of minor infractions and thus leading to higher eviction risk; or they may be more likely to have a “no second chances” policy for rental nonpayment. Further research is needed to investigate these possibilities, but whatever the case may be, the findings of this study present strong evidence that the eviction practices in PHAs with higher shares of Black residents are having a disparate impact on the residents of those buildings.

### 5.2 Limitations

This study has several limitations. We were only able to observe PHAs located in areas with robust coverage of evictions and HUD's building and *Picture of Subsidized Housing* data. Identifying eviction cases generated by PHAs was time-intensive and required merging multiple datasets acquired from different sources. This speaks not only to a lack of systematic collection of public housing evictions by HUD, the federal agency responsible for its administration, but also the difficulties of assembling our dataset from decentralized administrative files and court records. The PHAs in our sample covered a broad geography (Fig. D1) and had similar tenant population characteristics to PHAs excluded from our sample (Table A2); however, additional availability of data on eviction filings and PHA-level data from HUD could expand the generalizability of our results. Such data could also contain information on the reason the eviction case was filed and whether tenants were subsequently forced to vacate their units.

The dearth of data on how individual PHAs are managed also limited our ability to investigate the role that local policy may play in explaining variation in PHA filing rates. HUD requires that all PHAs maintain Admissions and Continued Occupancy Policies (ACOPs), but the agency does not consistently collect, review, or catalog these regulations. Although a handful of researchers have quantified ACOPs for several hundred PHAs (Curtis et al., 2013; Purtle et al., 2020), there exists no central database that would allow systematic investigation of tenant removal or eviction initiation policies. Without these data, we were unable to evaluate which PHA policies explain variation in PHA-level filing rates. Differences in procedures for handling past-due rent or policing of residents, including hiring of private security guards (Charles et al., 2019), may result in differential risk of eviction filings across PHAs.

We also lack a clear statement on eviction policy in public housing in the United States at both the federal and local (PHA) levels. We have documented that eviction filing rates vary widely across PHAs, with the share of filings against public housing tenants outpacing the share of tenants residing in public housing in many places, but we were not able to assess whether this variation, or relatively high filing shares, reflect the intentions of policies governing eviction actions of the PHA (locally) or HUD (nationally). The lack of clear policy for conditions that could (or should) result in an eviction filing against tenants by HUD, along with consistent documentation of implementation and enforcement of these policies across PHAs, raise critical but currently unanswerable questions about the management of these units. This lack of data and transparency also prevents researchers from evaluating the extent to which HUD meets its own standards (if any are in place) for shielding public housing tenants from threats of displacement, particularly those associated with inability to pay rent.

### 5.3 Policy implications

Our results have several implications for national housing policy. We demonstrated substantial variation in PHA filing rates both within and across states and significant associations between PHA filing rates and the prevalence of eviction filings in private rental housing in their service area. This suggests that PHAs are not operating independently of local housing markets and state- and county-level policies governing eviction behavior in private rental units.

Our findings also reveal the need to separate public housing from the larger legal and housing market forces in PHAs' service areas. Clear and uniform policies governing tenant dispute resolution, lease terminations, and eviction actions could reduce variation in eviction filing risk across PHAs. As eviction cases limit tenants' abilities to secure future rental housing, including eligibility for federal housing assistance (Desmond & Shollenberger, 2015), court-based eviction actions by PHAs compromise tenants' long-term rental stability and the ability to access affordable housing. Many PHAs in our sample had low eviction filing rates even under current HUD policy. Examining how these PHAs limit eviction filings could generate policy solutions that reduce the overall threat to residential instability in public housing.

The lack of comprehensive PHA eviction data limits researchers' ability to evaluate whether public housing fulfills its purpose of providing stable residence for low-income households and hinders legislators' ability to make evidence-based decisions about rental assistance and housing policy in the United States. To combat this, HUD could establish and enforce systematic collection of information on tenant characteristics and lease terminations in public housing across PHAs. PHAs providing aggregated counts for eviction filings and lease terminations in their annual reports of tenant characteristics would bring much needed transparency and accountability to their eviction dynamics. While the data presented here provide a first look, national eviction data from public housing is vital in establishing a complete picture of residential instability from subsidized public housing.

Larger shares of Black tenants were associated with higher PHA filing rates, net of other socio-economic characteristics of the tenant population. This association persisted when comparing PHAs located in the same county, indicating that this relationship is not attributable to local legal or housing context. The Fair Housing Act prohibits discriminatory rental practices on the basis of race (among other protected statuses), including eviction actions. Our findings indicate that disproportionately high eviction filing rates in PHAs with larger shares of Black renters warrant particular attention to ensure fair housing policies and prevent Black public housing tenants from the disparate impact of displacement (Desmond, 2012; Hartman & Robinson, 2003).

Our research indicates that oversight of eviction dynamics is particularly needed in larger PHAs. In 2016, the eviction filing rate in PHAs with at least 500 units was 17.5%, over three times the rate for PHAs with between 50 and 99 units (Fig. D4). Furthermore, results from the regression models consistently showed that larger PHAs were associated with higher filing rates, net of characteristics of the tenant population and surrounding service area. Determining why and with what consequences to their residents is an area of future research that could yield important policy recommendations, such as the implementation of eviction diversion programs (Network for Public Health Law, 2021; Witman Daley, 2020).

## CRediT authorship contribution statement

**Ashley Gromis:** Methodology, Formal analysis, Writing – original draft. **James R. Hendrickson:** Methodology, Formal analysis, Writing – original draft. **Matthew Desmond:** Conceptualization, Funding acquisition, Writing – review & editing.

## Declaration of competing interest

The authors have no competing interests to declare.

## Appendix A Supplementary Information

Supplementary Information Image 1

## Footnotes

3 These records are held by the Princeton University Eviction Lab. See Desmond, Gromis, Edmonds, Hendrickson, et al. (2018b) for additional details of record collection, cleaning, validation, and aggregation.

4 This was either the filing date (68% of cases) or first judgment date. When a case included both filing and judgment dates, the year was the same in 93.8% of cases.

5 We fit additional models removing multiple filings against the same household (SI Table C3); results did not differ.

6 Approximately 90% of cases listed only one tenant address. For cases with more than one address listed, we used the following ordered criteria to choose the primary address. First, we used the address associated with the initial filing, if present. If not, we used the address associated with a dismissal or forcible detainer judgment, as both of these judgments are likely to be issued against the disputed property. If no filing or judgment of this type was available, we assigned the modal address. If no modal address, we used the earliest address listed on the case. If there were still multiple address possibilities, we gave preference to in-state (as cases are unlikely to be filed across state lines) and geocode-able addresses (“different” addresses were often incomplete versions of another address listed on the case). Finally, we randomly choose an address from the possible addresses that met all preceding criteria. For additional information on how addresses were cleaned, see Desmond, Gromis, Edmonds, Hendrickson, et al. (2018b).

7 County is typically the smallest unit of geography for which courts release aggregated case filings.

8 We imposed the additional restriction that our data also represent no >114% of court-reported case counts, which may indicate either unreliable reporting from the courts or the presence of additional non-eviction landlord-tenant cases in our data. We tested the robustness of this threshold by restricting the sample to counties with at least 90% (but no >110%) coverage or a difference of less than five cases and loosening the sample to counties with at least 80% (but no >120%) of coverage or a difference of <15 cases. Results did not differ substantially in the analyses (available from authors upon request).

9 Complete information was available for 95.5% of addresses.

10 <1% of addresses in the building and unit files could not be matched to the *Housing Authority Profiles*, suggesting strong correspondence across data files. To link addresses to a project name, we matched the project identifiers to *HUD Open Data*, which contained a listing of HUD buildings and associated project names. A higher portion of our addresses (14.9%) could not be assigned a project name.

11 The missing FIPS codes largely reflected small towns that existed as CDPs in the 2000 Census but were no longer considered independent CDPs in the 2010 Census and were not assigned FIPS codes.

12 We considered city-level PHAs to have validated, adequate coverage if all counties that contained at least 10% of the CDP area had validated, adequate coverage. To determine shared area, we used the geographic correspondence files for 2010 Census CDPs and counties available from the Geographic Correspondence Engine maintained by the Missouri Census Data Center (Missouri Census Data Center, 2018). Most CDPs were fully contained within one county.

13 We conducted robustness checks in which the threshold for percent occupied units reporting data was higher (90%) and substantially lower (50%). The choice of threshold did not substantially alter our findings (results available upon request).

14 As you cannot take the natural log of 0, we added a small constant (0.01) to the filing rate before logging. To ensure that the choice of this small constant did not affect our results, we performed the same analyses using smaller (0.001) and larger (0.1) values; results did not differ substantially (available from authors upon request).

15 The descriptive statistics of this sub-sample did not substantively differ from those reported in Table 1 (table available upon request).

16 We calculated PHA filing rates using occupied units as the denominator to maintain comparability with private filing rates, which are based on occupied renter households; however, if greater eviction filings led to more turnover and vacancies in some PHAs, this may inflate the filing rate. To test this we also calculated filing rates using total public housing units as the denominator. Rates did not change significantly (SI Figure D5).

17 We conducted a robustness check on these results by imposing the more restrictive requirement that cities must have 100% of their area contained within a county to be included in the model with county-level fixed effects. The results did not differ substantively (available upon request).

18 Our data do not permit us to identify whether Black public housing tenants were individually at higher risk of eviction threat.
