# Supplementary material for: Eviction from public housing in the United States
Source: Cities. 2022 Aug;127:103749. doi: 10.1016/j.cities.2022.103749 (PMC9207190; doi:10.1016/j.cities.2022.103749)
Supplement: Supplementary file 1 — Supplementary Information [file mmc1.docx]

**Supplementary Information**

Eviction from Public Housing in the United States

**Contents**

A. Identification and Assignment of Public Housing Cases

B. Public Housing Waitlist Measures

C. Supplementary Tables

D. Supplementary Figures

**A. Identification and Assignment of Public Housing Cases**

**A.1. Identifying Public Housing Cases**

Eviction records neither contain indicators identifying cases filed by PHAs nor does HUD require that PHAs track or report the filing of eviction cases in local courts. Identifying cases filed against public housing tenants required us to match the PHA or project names and addresses included in the HUD data to plaintiff names and tenant (defendant) addresses in the eviction records. We used four methods to accomplish this:

(a) Matching common terms in PHA names to plaintiff names

(b) Matching PHA building entrance and unit addresses in the HUD files to tenant addresses

(c) Matching PHA and public housing development names in the HUD files to plaintiff names

(d) Extrapolating matched plaintiff names and tenant addresses in the same geographic area

**A.1.a. Common PHA Terms**

We used regular expressions to search for common terms in PHA names (e.g., “housing authority,” “housing agency,” “redevelopment commission,” “county housing”) in plaintiff names in the eviction records. The list of terms is included in Table A1. We developed the full list of terms by manually reviewing PHA names included in the HUD data files and plaintiff names on eviction filings. We used wildcard characters in many of the search strings to account for abbreviations and typographical errors common in administrative data. We reviewed and revised terms to match plaintiff names both within and across states.

| **Geographic Area** | **Search String** |
| --- | --- |
| All states | ("HOUSING" & "AUTH") \| "^H ?A OF " \| "^(COUNTY\|CITY\|PUBLIC) HOUSING$" \| "PI?UL?B[LICDS]+ HOUSING" |
|  | "([^A-Z]\|^)H[OUSEING ]+ AU(T\|$)" & !"THROUGH HIS\|AUTO S\| LLC\|HIGGINS" & length > 10 |
|  | "([^A-Z]\|^)H[OUSEING ]+AUTH" & !"THROUGH HIS\| LLC" |
|  | "(^\|[^A-Z])(H[OU]+[SIN]+G\|HOUS\|HSI?N?G\|H[OSUI]+ING\|[A-Z0-9]OUSING)" & "(^\|[^A-Z])(AUU?[TH]([HORITY]\| \|$)\|A[A-Z0-9/-]+OT?RITY)" & !" INC" |
|  | "HOU[SING ]+AU" & !"AUTUMN" |
|  | "(^\|[^A-Z])(H[OU]+[SIN]+G\|HOUS\|HS(IN)?G\|H[OSUI]+ING\|[A-Z0-9]OUSING)" & "(^\|[^B-Z])PUBLIC" |
|  | "HO[A-Z]+NG" & "(^\|[^A-Z])(AUU?[TH]([HORITY]\| \|$)\|A[A-Z0-9/-]+ORITY)" & !"HOLDING" |
|  | **county_name** + ",? CO(UNTY)? HOUS" & !" (INC\|LLC\|LTD)( \|$)" |
|  | **str2_county_name** + "[A-Z\. ]* CO(UNTY)? HOUS" & !" (INC\|LLC\|LTD\|PARTNERSHIP)( \|$)" |
|  | "^" + **str2_county_name** + "[A-Z\. ]* COUNTY$" & !"CAC " |
|  | **county_name** + " ?C?O?U?N?M?T?R?Y? (HRA\|[CE]DA\|[MR]? ?H ?A\|HOUS?I?N?G?)$" |
|  | "(^\|[^A-Z])(H[OU]+[SIN]+G\|HOUS\|HS(IN)?G\|H[OSUI]+ING\|[A-Z0-9]OUSING)" & "(CITY\|COUNTY) OF" |
|  | "REDEV" & "( \|^)(AUU?[TH]([HORITY]\| \|$)\|A[A-Z0-9/-]+ORITY)" |
|  | "(^\|[^A-Z])(H[OU]+[SIN]+G\|HOUS\|HS(IN)?G\|H[OSUI]+ING\|[A-Z0-9]OUSING\|HOU$\|AGENCY)" & "REDEV\|COM+ISSION" & !"WINTER PARK" |
|  | "(^\|[^A-Z])(H[OU]+[SIN]+G\|HOUS\|HS(IN)?G\|H[OSUI]+ING\|[A-Z0-9]OUSING)" & " REG[IONAL]* " & !" (INC\|LLC)( \|$)" |
|  | " REG[IONAL' ]+HOUS" & !" (LLC\|INC)" |
|  | "(^\|[^A-Z])(H[OU]+[SIN]+G\|HOUS\|HS(IN)?G\|H[OSUI]+ING\|[A-Z0-9]OUSING)" & "CITY\|COUNTY\|REGION\|MUNIC" & "AGENCY" |
|  | "(CO(UNTY)?\|CI?TY) H?O?U?S?I?N?G? ?AU[TH][TH]" & !"MGT" |
|  | "^(THE )?CO(UNTY)? OF " + **county_name** + "$" \| "^" + **county_name** + " CO(UNTY)?$" |
|  | "^(THE )?CITY OF " & !" (TAX\|HEALTH\|INC\|LLC\|AIRPORT\|WATER( \|$)\|ZONING)" |
|  | "^" + **city_name** + " CITY$" |
| Louisiana | "PARISH HOUSING" \| "PARISH OF [A-Z ]+ HOUSING" |
| Alabama & Montana | "HO[US][US]" & " B[OAR]+D" |
| Florida | "^((M[IA][AI]\|METO?R)[A-Z ]*[ -])?DADE? (COUNTY\|HOUSING AGENCY)$" |
| Georgia | "^JACKSON CO(UNTY)? HOUSING$" |
| Iowa | " (HOUSE?(IN?G)? AGENCY\|MULTI CO)" & !"INC" |
| Indiana | " HOU[SEING]* (AGENCY\|NEIGHBORHOOD)" & !"INC" |
| Michigan | ("HOU[SRING ]+COM([MI]\|$)" \| "COUNTY HOUSING") & !"COMMUN" |
| Nebraska | ("HOUS[EINGT ]*AG" \| "^COUNTY OF") |
| New Mexico | "HOUS" & "COUNTY\|REG\|AGENCY” |
| Oregon | ("HOUS[A-Z0-9 ]+AND ?COMM" \| "OREGON( DEPARTMENT OF)? HOUS") & !"PARTNERSHIP" |
| Tennessee | "HOI?U?[SUING]+ " & "AGEN" |
| Virginia | "REDE?V\|REDE?V?E?L" & "HOU\|AUTH" |
| New York | ("^NYCHA" \| "NYCHA$") |

Note: **county_name** = county name where case was filed, **str2_county_name** = first 2 letters of county name where case was filed, **city_name** = city name associated with case address.

**Table A1.** Search strings used to identify common PHA terms in plaintiff names

**A.1.b. PHA Addresses**

We performed an inexact, probabilistic match between tenant addresses in the eviction records and PHA addresses in the HUD data within the same state using the reclink program (Blasnik, 2010) in Stata (version 15.0). This program calculates a similarity score between tenant addresses in the eviction records and PHA addresses (building entrance or unit) included in the HUD files. The similarity scores ranged from 0 (no matching characters between the eviction record and PHA address) to 1 (an exact match between the eviction record and PHA address). We retained a match if the similarity score was 1 (indicating an exact match between the addresses) or the addresses shared the same street number, city name, and met at least one of the following criteria:

(1) A reclink similarity score equal to or greater than 0.5 and 75% or more text shared between the tenant and PHA addresses. We calculated the percentage of shared text in three steps. First, we separated the tenant street address strings into pieces using whitespace as the delimiter (e.g., 123 Main Street becomes “123,” “Main,” “Street”). Second, we used regular expressions to count how many of these separated pieces appeared in the PHA street address. Third, we created a percentage of shared text by dividing the total number of tenant address pieces by the number that the regular expressions marked as appearing within the PHA street address. We repeated these same steps to calculate the percentage of string pieces of the PHA street address appearing in the tenant eviction record address. If the reclink similarity score was greater than or equal to 0.5 and either of these two percentages of shared text were 75% or greater, we retained the record match.

(2) A reclink similarity score of at least 0.9, at least 66% of shared text between the tenant and PHA addresses, and the plaintiff name contained any common PHA terms identified in section 1.a. above.

(3) A reclink similarity score of at least 0.93 and the plaintiff name contained any common PHA terms identified in section 1.a. above.

We excluded any matches in which the eviction filing occurred outside the dates the PHA address was listed as active in the HUD data files.

**A.1.c. PHA and housing development names**

We performed a second probabilistic reclink match between plaintiff names in the eviction records and PHA and development names in the HUD file within the same state. Matching the plaintiff names was more difficult than addresses for two reasons. First, names of some private rental market housing complexes were very similar to PHA names (e.g., “Hudson Country Apts” and “Hudson County HA”). Many PHAs also share similar naming conventions, which we exploited in the regular expression text searches to identify public housing cases (e.g., “Hudson City HA” and “Hudson County HA”). This makes it difficult to definitively assign cases to a PHA.^^[[1]](#footnote-1)^^ For these reasons, we only retained matches that met stricter criteria than those used for matching addresses in Section 1.b.:

(1) The PHA or development name and the plaintiff name were an exact match (a reclink similarity score of 1).

(2) The PHA development name and plaintiff name had a reclink similarity score of 0.95 and at least one eviction record sharing the same PHA development and plaintiff names was also matched by address (Section 1.b).

**A.1.d. Extrapolation of previous matches**

Finally, we extrapolated from the matches identified by eviction record and PHA address (Section 1.b.) and PHA or development and plaintiff name (Section 1.c.). If both the plaintiff name and address on an eviction record matched a PHA entry in the HUD data, we assigned all cases with the same plaintiff name located in the same city to that PHA. Additionally, if a unique plaintiff name had at least 50% of associated property addresses in the eviction records matched to a PHA in Section 1.b., we assigned the remaining eviction records associated with that plaintiff name to the same PHA.

**A.1.e. Identification summary**

Using these four methods, we identified just under 1 million cases (N=979,988) filed by PHAs. Nearly half (48.8%) of the cases were identified by addresses and common PHA terms appearing in the plaintiff name (Column 1, Table A2). An additional 10.4% of cases matched common PHA terms, a known PHA address, and the PHA name. A matched address was the only PHA indicator for 19.4% of the cases. This indicates that searching for PHA names among plaintiffs in eviction records is not sufficient to identify the population of public housing cases. This could be due to variation in how PHA names are entered in eviction records or listing the property manager or other official as the plaintiff when filing a case. Only 2.2% of cases were identified by extrapolating from previous matches. The remaining 19.3% of cases were either identified by common PHA terms alone (Section 1a) or some combination of the address, name, or common PHA term indicators.

|  | **(1)** | | **(2)** | | |
| --- | --- | --- | --- | --- | --- |
| **Identification Method** | **All Cases** | | **Cases in**  **Analytic Sample** | | |
|  | **N** | **%** | | **N** | **%** |
| *Single indicator* |  |  | |  |  |
| Common PHA terms (Section 1a) | 112,890 | 11.52 | | 32,657 | 9.93 |
| Address (Section 1b) | 190,036 | 19.39 | | 63,655 | 19.36 |
| PHA/development name (Section 1c) | 16,755 | 1.71 | | 2,875 | 0.87 |
|  |  |  | |  |  |
| *Multiple indicators* |  |  | |  |  |
| Address & common PHA terms | 477,937 | 48.77 | | 152,482 | 46.37 |
| PHA/development name & common PHA terms | 25,692 | 2.62 | | 11,975 | 3.64 |
| Address & PHA/development name | 33,887 | 3.46 | | 7,109 | 2.16 |
| All three indicators | 101,749 | 10.38 | | 51,552 | 15.68 |
|  |  |  | |  |  |
| *Extrapolation of matches (Section 1d)* |  |  | |  |  |
| Records with plaintiff names and addresses that matched PHA name and address in the same city | 1,952 | 0.20 | | 675 | 0.21 |
| 50% plaintiff records matched PHA address | 18,983 | 1.94 | | 5,826 | 1.77 |
| 50% plaintiff records matched PHA address & common PHA terms | 107 | 0.01 | | 39 | 0.01 |
| Total | 979,988 | 100.00 | | 328,845 | 100.00 |

**Table A2.** Identification methods for public housing cases

The second column of Table A2 shows the distribution of identification methods of only the cases for PHA-years included in the analytic sample (N=328,845 cases across 1,243 PHAs). There is little substantive difference between the distributions of case identification methods for the full set of cases versus those included in the analytic sample, suggesting that the sample cases are representative of how public housing cases generally appear in the eviction court records.

Our address linkages did not contain information on apartment or unit numbers due to variation in how this information was represented (and whether it was included) in eviction records. Some HUD redevelopment programs (e.g., HOPE VI, Choice Neighborhoods) create the conditions for private rental units to be contained in the same complex as public housing units. As a result, some unknown proportion of the properties in the cases matched by address only may be non-public housing. This should not be an issue for the cases matched by common PHA terms or PHA or development name (or combinations of these matches with known PHA addresses) as we would not expect the PHA to be listed as the plaintiff for non-public units. To ensure this the potential over-inclusion of non-public units was not significantly affecting our calculation of PHA filing rates or analyses, we ran additional models that excluded any PHA-year observations in which more than 5% of cases were matched to the PHA by address only. Neither the filing rates (Figure D7), nor the findings from the regression analyses (Table C4) differed substantively.

**A.2. Assigning public housing cases to PHAs**

Calculating PHA filing rates required us to determine the PHA responsible for each of the cases we identified as filed against public housing tenants. This was straightforward when both the plaintiff name and tenant address on a case matched a PHA listing in the HUD data (49.3% of all cases, 61.7% of cases in the analytic sample) (Table A3). We assigned the same PHA to cases filed under the same plaintiff names in the same cities (9.1% of all cases, 11.5% of cases in analytic sample).

|  | **(1)** | | **(2)** | |
| --- | --- | --- | --- | --- |
| **Assignment Criteria** | **All Cases** | | **Cases in**  **Analytic Sample** | |
|  | **N** | **%** | **N** | **%** |
|  |  |  |  |  |
| (1) Linked by address, plaintiff and PHA name matched | 483,283 | 49.32 | 202,831 | 61.68 |
| (2) Same plaintiff name & city as cases identified in (1) | 89,179 | 9.10 | 37,797 | 11.49 |
| (3) Linked by address, plaintiff and PHA name did not match | 319,893 | 32.64 | 71,909 | 21.87 |
| (4) Same plaintiff name, street name, and city as (3) | 19,216 | 1.96 | 5,078 | 1.54 |
| (5) Same plaintiff name, first word in street name, and city as (3) | 5,465 | 0.56 | 688 | 0.21 |
| (6) Same plaintiff name and city as (3), only one PHA in city/county | 16,579 | 1.69 | 2,261 | 0.69 |
| (7) Same plaintiff name as (3), plaintiff name linked to only one PHA | 15,203 | 1.55 | 1,285 | 0.39 |
| (8) Cased assigned by PHA or development name | 4,639 | 0.47 | 5,769 | 1.75 |
| (9) Plaintiff name matched 1 active PHA in city/county | 8,146 | 0.83 | 1,173 | 0.36 |
| (10) Plaintiff name matched 1 active PHA in state | 1,003 | 0.10 | 54 | 0.02 |
| Unassigned | 17,382 | 1.77 |  |  |
| Total | 979,988 | 100.00 | 328,845 | 100.00 |

**Table A3.** Methods of assigning public housing cases to PHA

It was also straightforward to determine the PHA when the case address matched a PHA address, even if the plaintiff name on the case was not the same as the PHA name associated with the address in the HUD data (32.6% of all cases, 21.8% of cases in analytic sample). This often occurred when the plaintiff names were entered as unspecific housing authorities (e.g., “The Housing Authority” or “Housing Agency”). We then assigned cases to the same PHA if filed under the same plaintiff name and the rental property was located on the same street in the same city as a case that matched a known PHA address (Criteria 4 and 5 in Table A3). Cases sharing the same plaintiff name were also assigned to the same PHA if there was only one active PHA in the city in which the disputed property was located (Criteria 6) or there was only one distinct PHA associated with the plaintiff name (Criteria 7). Collectively, we were able to determine the PHA for an additional 5.8% of total cases identified as filed by PHAs (2.8% of cases in analytic sample).

For the remaining cases, we determined the PHA responsible for the filing by the plaintiff name alone. If the case had been identified as public housing by definitively matching the plaintiff name to a known PHA or development name in the HUD data (Section 1c), the case was assigned to the matched PHA (0.5% of total cases; 1.8% of cases in analytic sample). Finally, we compared plaintiff names to the names of all PHAs active in the same city and county in which the disputed property was located. If there was a clear, singular match between the plaintiff name and a PHA name, we determined that PHA to have been responsible for the filing (0.8% of all cases; 0.4% in analytic sample). We repeated this same process for all active PHAs in the state in which the disputed property was located if we were unable to determine the PHA through the previous methods (0.1% of all cases; less than 0.1% of cases in analytic sample).

We were unable to determine the PHA responsible for 17,382 filings identified as coming from public housing (1.8% of total cases). It is almost impossible to discern how many, if any, rightfully belong to the PHAs included in the analytic sample. Most of these cases could not be assigned to a PHA for one of three reasons:

(1) The plaintiff name appeared to list a PHA that was not included in the HUD data.

(2) The plaintiff name was too ambiguous to definitively determine the PHA.

(3) The properties were not located in areas with known PHAs or near other properties listed on cases able to be assigned to PHAs.

Even if all these cases belonged to PHAs included in the analytic sample, which would be unlikely in (1) and (3) above, they would represent only 5.3% of all public housing cases. This suggests that they would not substantially increase the overall prevalence of eviction filings in the PHAs in the analytic sample.

**A.3.** **PHA service areas**

PHAs manage housing units in service areas at different levels of geography. Many PHAs operate within cities (e.g., Housing Authority of the City of Los Angeles, New York City Housing Authority), while others serve counties (e.g., Allegheny County Housing Authority) or entire states (e.g., Hawaii Public Housing Authority). To compare PHA filing rates to those of non-public housing in the same geographic area, we needed to know the geographic boundaries of the service areas. Unfortunately, the data obtained from HUD did not include information on the service areas managed by the PHAs.

To determine the PHA service areas, we compared the PHA name to the city, county, and state names in which their associated building entrance and/or unit addresses were located. PHAs could be assigned to five mutually exclusive levels of geography:

(1) City: The PHA name contained the city name or the string “city” or “town” (N=2,535).

(2) County: The PHA name contained the county name or the string “county” (N=447).

(3) City and County: The PHA name matched the name of both the city and county and did not contain additional text indicating “city” or “county” (N=209). This only occurred when a city and county shared the same name (e.g., Philadelphia is located in Philadelphia County, PA). For some places, like Philadelphia, the city and county boundaries are almost spatially indistinguishable. For others, there is a more distinct difference in the amount of area occupied by the city or county.

(4) Regional: The PHA name contained multiple counties or contained the strings similar to “regional” or “area” (N=56).

(5) State: The PHA name contained only the name of the state (N=10).

We were unable to determine the service areas for 1.2% of PHAs (N=40). We excluded PHAs from the analysis that operated at the regional or state levels or had an unknown service area (N=106). PHAs that were categorized as “City and County” were considered to operate at the city level in the primary analyses. We assigned city-level boundaries as 77.8% (N=2,535) of the PHAs with known service areas (N=3,257) operated at the city level. We conducted robustness checks that assigned the “City and County” PHAs the county boundaries and county-level FIPS codes instead, but results did not differ substantively (results available upon request).

**B. Public Housing Waitlist Measures**

Differences in how households are placed in public housing units across PHAs complicate comparisons between tenant populations in PHAs and private rental units in the PHA service area. Beyond federal income eligibility requirements, PHAs have considerable discretion in prioritizing particular at-risk groups for placement into public housing (Martinez & Sard, 2000; Leopold, 2012). This can result in tenant populations that are highly selective, variable across PHAs, and not representative of residents of similarly sized housing complexes in low-income areas. In 2012, HUD conducted a survey of how PHAs managed subsidized housing waitlists, including preferences for placement in public housing units. HUD makes these data publicly available at <https://www.huduser.gov/portal/datasets/pha_study.html>. We downloaded and matched these data with the PHAs in our sample to create supplementary measures of tenant population characteristics that may be associated with likelihood of eviction filings.

The HUD survey recorded responses for 3,210 of a total 3,988 active PHAs in 2012 (an 80.5% response rate). We dropped 659 PHAs that reported no public housing units (and therefore had no data to report on public housing waitlist preferences), 11 PHAs that reported active public housing units but did not provide data on public housing units, and an additional 3 PHAs located in outlying U.S. territories. We then merged the 2,537 remaining PHAs to our sample data file. We were unable to match 92 PHAs in our sample data file because the PHAs had unknown geographic service areas (N=13), operated at the state or regional level (N=37), operated at the city level but could not be matched to a known CDP FIPS code (N=35), or were not included in any of our other HUD data files (N=7). Of the 1,243 PHAs included in our analytic sample, 1,013 (81.5%) were represented in the HUD waitlist survey data, very close to the overall PHA response rate reported by HUD. We excluded an additional 11 PHAs from the final analyses due to incomplete data on the public housing waitlist measures.

The survey asked about 10 possible unlimited public housing waitlist preferences. We created 4 combined indicators of the possible preferences:

1. Severe socio-economic (SES) disadvantage – tenants who had been homeless, severely rent burdened, or living in substandard housing

2. Displacement – tenants who were displaced due to public action or natural disaster

3. Vulnerable groups – veterans, persons with disabilities transitioning out of nursing homes or institutions, victims of domestic violence, families referred by public child welfare agencies for family reunification, or youth transitioning out of foster care

4. Employed – tenants or families who were working^[[2]](#footnote-2)^

All indicators were constructed as dummy variables with “1” indicating that at least one of the listed groups was given preference on the public housing waitlist. The survey did not ask about preferences for elderly or disabled tenants. Rather, there was a separate question asking if all public housing units were exclusively reserved for elderly or disabled tenants. For this reason, we created a separate indicator for elderly/disabled tenants. This indicator represents a much more selective public housing population as the wording implies that *only* elderly or disabled tenants may reside in the public housing units, as opposed to indicating preferential status on the waitlist.

The inclusion of the waitlist variables altered some of the results in the full sample (first column of Table B1). None of the waitlist preference indicators were significantly associated with PHA filing rate, although their inclusion did eliminate the significant association between the PHA filing rate and both the percent tenant households with children and households with extremely low income. The other significant associations—percent Black tenant population, senior (aged 65 or older) householders, size of the PHA, and the filing rate in private rental units in the PHA service area—remained unchanged. Removing percent senior householders from the model resulted in a significant negative association between the indicator for all senior/disabled housing and PHA filing rates, which we expected given the association between percent senior householders and PHA filing rates. We also did not find significant effects of waiting list preferences when restricting the sample to only those PHAs located in counties with at least two PHA observations in the same year (second column of Table B1). Significant associations between percent Black tenant population and PHA filing rate also remained after accounting for waitlist preferences in the within-county model. That there was no relationship between preference for severely economically disadvantaged households and the PHA filing rates suggests that over-selection of severely economically disadvantaged households into some PHAs does not explain variation in filing rates across PHAs.

| **Variable** |  | **Full Sample** | | | **Within-County Sample** | | |
| --- | --- | --- | --- | --- | --- | --- | --- |
|  |  | **Coeff.** | **SE** | **Sig.** | **Coeff.** | **SE** | **Sig.** |
| *Public Housing Authority* |  |  |  |  |  |  |  |
| % Black tenants (in 10%) |  | 0.103 | 0.036 | 0.004** | 0.142 | 0.056 | 0.012* |
| % Hispanic tenants (in 10%) |  | -0.061 | 0.049 | 0.209 | -0.003 | 0.078 | 0.972 |
| % Hhs with children (in 10%) |  | 0.069 | 0.047 | 0.145 | 0.097 | 0.081 | 0.231 |
| % Female-headed hhs (in 10%) |  | -0.085 | 0.052 | 0.102 | -0.067 | 0.078 | 0.393 |
| % Extremely low income (in 10%) |  | 0.058 | 0.033 | 0.075 | 0.013 | 0.050 | 0.793 |
| % Hhs aged 65+ (in 10%) |  | -0.176 | 0.046 | 0.000*** | -0.124 | 0.078 | 0.109 |
| Months on waiting list |  | -0.005 | 0.003 | 0.131 | -0.008 | 0.004 | 0.067 |
| Waitlist preferences |  |  |  |  |  |  |  |
| All senior/disabled |  | -0.321 | 0.173 | 0.064 | -0.374 | 0.278 | 0.179 |
| Severe SES disadvantage |  | -0.313 | 0.176 | 0.075 | -0.462 | 0.264 | 0.080 |
| Displacement |  | 0.370 | 0.199 | 0.063 | 0.274 | 0.308 | 0.374 |
| Vulnerable groups |  | -0.016 | 0.194 | 0.935 | -0.008 | 0.258 | 0.976 |
| Employed |  | -0.237 | 0.230 | 0.301 | -0.596 | 0.321 | 0.064 |
| PHA size |  |  |  |  |  |  |  |
| 1 - 99 units |  | -1.876 | 0.198 | 0.000*** | -1.889 | 0.302 | 0.000*** |
| 100 - 299 units |  | -0.772 | 0.186 | 0.000*** | -1.042 | 0.274 | 0.000*** |
| 300 - 499 units |  |  |  |  |  |  |  |
| 500 - 999 units |  | 0.171 | 0.192 | 0.374 | -0.133 | 0.317 | 0.674 |
| 1000+ units |  | 0.605 | 0.195 | 0.002** | 0.412 | 0.293 | 0.160 |
| *Service Area* |  |  |  |  |  |  |  |
| % Black pop. (in 10%) |  | -0.082 | 0.069 | 0.234 | -0.059 | 0.128 | 0.644 |
| % Hispanic pop. (in 10%) |  | -0.090 | 0.068 | 0.182 | -0.171 | 0.124 | 0.167 |
| % Female-headed hhs |  | 0.005 | 0.015 | 0.714 | -0.010 | 0.024 | 0.676 |
| % Families in poverty |  | 0.001 | 0.010 | 0.883 | 0.011 | 0.016 | 0.494 |
| % Unemployed |  | 0.009 | 0.014 | 0.521 | 0.027 | 0.022 | 0.209 |
| % Renting hhs |  | 0.008 | 0.007 | 0.224 | 0.002 | 0.011 | 0.855 |
| % Hhs with children |  | 0.015 | 0.010 | 0.129 | 0.014 | 0.019 | 0.470 |
| Property value (in $1000s) |  | 0.001 | 0.001 | 0.412 | 0.000 | 0.002 | 0.902 |
| Eviction Filing rate (logged) |  | 0.116 | 0.028 | 0.000*** | 0.106 | 0.045 | 0.019* |
| Constant |  | -1.086 | 0.599 | 0.070 | -2.083 | 1.798 | 0.247 |
| R^2^ (overall) |  | 0.570 | | | 0.636 | | |
| N |  | 6,402 (1,002 PHAs) | | | 3,533 (558 PHAs) | | |

Note: *** p< 0.001; ** p< 0.01; * p< 0.05. Fixed effects for states (for the full sample) or counties (for the within-county sample) and years were included in the model but not shown in the table due to space considerations.

**Table B1.** Effects of public housing waitlist preferences, 2006-2016

There are several limitations to these results. First, we have matched cross-sectional data HUD collected from PHAs in 2012 to a longitudinal sample of PHA population characteristics and filing rates. The implicit assumption is that waitlist preferences have remained relatively stable over time. It is not possible to investigate the validity of this assumption without additional data. Second, while our ability to match our sample PHAs to the waitlist data appeared roughly consistent with the overall response rate for the survey reported by HUD, we have no means of assessing whether the responses from these PHAs are representative of the PHAs who did not respond (or were not surveyed by HUD). Third, we made the assumption that, after providing non-missing responses to the previous questions about overall waitlist preferences, missing data regarding whether individual groups were given preference indicated no preference for that group.^[[3]](#footnote-3)^ We could find no other skip patterns or clear patterns of missingness in previous questions that otherwise explained the missing values. If these missing values actually indicated that the PHA was not asked these questions, or some other form of non-response, this may alter the results presented here. For these reasons, we presented these results as supplementary models rather than in the main text.

This limited insight into selection of tenants into public housing supports our primary findings. Waitlist preferences for public housing units neither showed substantial associations with PHA filing rates, nor explained significant relationships between tenant population socio-demographic characteristics or filing rates in private rentals in the PHA service area and PHA filing rates. These results provide additional evidence that socio-economic disadvantage was not the primary explanation for filing prevalence in public housing. While public housing may eliminate some of the financial burden associated with housing costs in the private rental market, weakening direct associations between poverty and risk of eviction in public housing, it does not mitigate overall risk of eviction for its tenants.

**C. Supplementary Tables**

| **Year** | **Data Source** |
| --- | --- |
| 2006 | 2005-2009 5-Year ACS |
| 2007 |  |
| 2008 |  |
| 2009 |  |
| 2010 | 2010 Census (short form) & 2008-2012 5-Year ACS (long form) |
| 2011 | 2011-2015 5-Year ACS |
| 2012 |  |
| 2013 |  |
| 2014 |  |
| 2015 |  |
| 2016 | 2014-2018 5-Year ACS |

**Table C1.** Yearly assignment of Census and American Community Survey (ACS) data for socio-demographic characteristics of surrounding area

|  | **Analytic Sample (N=7,821)** | | | | | | | **Excluded from Analytic Sample (N=26,763)** | | | | |
| --- | --- | --- | --- | --- | --- | --- | --- | --- | --- | --- | --- | --- |
| Variable | N | Mean | Std. Dev. | Median | Min | Max | N | Mean | Std. Dev. | Median | Min | Max |
| *Public Housing Authority* |  |  |  |  |  |  |  |  |  |  |  |  |
| Total units | 7,821 | 311.68 | 783.43 | 100.00 | 12.00 | 10,490.00 | 24,794 | 372.12 | 3,755.64 | 103.00 | 1.00 | 179,714.00 |
| Occupied units | 7,821 | 289.51 | 713.56 | 97.00 | 10.95 | 10,079.30 | 24,677 | 347.04 | 3,652.96 | 99.00 | 0.00 | 174,456.70 |
| % Black tenants | 7,821 | 33.14 | 36.18 | 15.00 | 0.00 | 100.00 | 24,382 | 27.28 | 33.30 | 9.00 | 0.00 | 100.00 |
| % Hispanic tenants | 7,821 | 8.17 | 17.56 | 1.00 | 0.00 | 100.00 | 24,382 | 10.07 | 20.69 | 1.00 | 0.00 | 100.00 |
| % Households with children | 7,821 | 37.11 | 22.39 | 38.00 | 0.00 | 100.00 | 23,446 | 36.76 | 21.05 | 37.00 | 0.00 | 100.00 |
| % Female-headed households | 7,821 | 74.21 | 11.03 | 75.00 | 19.00 | 100.00 | 24,328 | 73.25 | 10.48 | 74.00 | 8.00 | 100.00 |
| % Households extremely low income | 7,821 | 63.17 | 16.47 | 65.00 | 0.00 | 100.00 | 24,300 | 62.38 | 16.12 | 64.00 | 0.00 | 100.00 |
| % Householders aged 65+ | 7,821 | 34.66 | 21.43 | 29.00 | 0.00 | 100.00 | 24,328 | 36.10 | 21.12 | 31.00 | 0.00 | 100.00 |
| Months on waiting list (mean) | 7,821 | 10.70 | 13.92 | 6.00 | 0.00 | 255.00 | 23,369 | 11.28 | 29.48 | 6.10 | 0.00 | 2,553.00 |
| Size |  |  |  |  |  |  |  |  |  |  |  |  |
| 1 - 99 units | 3,081 | 0.39 |  |  | 0.00 | 1.00 | 9,211 | 0.37 |  |  | 0.00 | 1.00 |
| 100 - 299 units | 1,941 | 0.25 |  |  | 0.00 | 1.00 | 6,246 | 0.25 |  |  | 0.00 | 1.00 |
| 300 - 499 units | 729 | 0.09 |  |  | 0.00 | 1.00 | 2,647 | 0.11 |  |  | 0.00 | 1.00 |
| 500 - 999 units | 817 | 0.10 |  |  | 0.00 | 1.00 | 2,907 | 0.12 |  |  | 0.00 | 1.00 |
| 1000+ units | 1,253 | 0.16 |  |  | 0.00 | 1.00 | 3,928 | 0.16 |  |  | 0.00 | 1.00 |
| *Service Area* |  |  |  |  |  |  |  |  |  |  |  |  |
| % Black population | 7,821 | 14.69 | 18.75 | 5.77 | 0.00 | 95.48 | 26,763 | 12.09 | 17.46 | 4.07 | 0.00 | 98.36 |
| % Hispanic population | 7,821 | 9.89 | 15.09 | 3.93 | 0.00 | 99.52 | 26,763 | 11.81 | 17.79 | 4.39 | 0.00 | 99.84 |
| % Female-headed households | 7,821 | 13.91 | 5.78 | 12.86 | 1.83 | 43.86 | 26,763 | 13.61 | 5.68 | 12.67 | 0.70 | 47.05 |
| % Families in poverty | 7,821 | 14.68 | 7.29 | 13.68 | 0.00 | 49.58 | 26,761 | 14.25 | 7.56 | 13.11 | 0.00 | 61.69 |
| % Unemployed | 7,821 | 8.44 | 4.04 | 7.85 | 0.00 | 32.83 | 26,761 | 8.13 | 3.89 | 7.60 | 0.00 | 33.50 |
| % Renting households | 7,821 | 35.28 | 11.36 | 33.99 | 6.77 | 81.61 | 26,763 | 34.94 | 11.39 | 33.96 | 1.56 | 82.83 |
| % Households with children | 7,821 | 31.07 | 5.84 | 30.86 | 9.19 | 69.76 | 26,763 | 31.89 | 6.72 | 31.34 | 6.53 | 82.96 |
| Property value (median, in $1000s) | 7,821 | 126.01 | 78.47 | 105.40 | 28.00 | 997.01 | 26,763 | 138.47 | 107.21 | 102.85 | 25.70 | 1,108.37 |

Note: All measures calculated annually and averaged across years in sample. PHA characteristics calculated from the HUD *Picture of Subsidized Housing*. Surrounding area characteristics calculated using data from the Census and ACS as shown in Table C1. We cannot report eviction case filings or filing rates for the out-of-sample PHA-years due to lack of reliable eviction data for the counties in which many of these PHAs were located.

**Table C2.** Descriptive statistics for PHA-years, by inclusion in analytic sample

.

| **Variable** |  | **Full Sample** | | | **Within-County Sample** | | |
| --- | --- | --- | --- | --- | --- | --- | --- |
|  |  | **Coeff.** | **SE** | **Sig.** | **Coeff.** | **SE** | **Sig.** |
| *Public Housing Authority* |  |  |  |  |  |  |  |
| % Black tenants (in 10%) |  | 0.086 | 0.031 | 0.006^**^ | 0.157 | 0.049 | 0.001^**^ |
| % Hispanic tenants (in 10%) |  | -0.088 | 0.045 | 0.051 | 0.009 | 0.062 | 0.885 |
| % Hhs with children (in 10%) |  | 0.091 | 0.042 | 0.030^*^ | 0.083 | 0.066 | 0.208 |
| % Female-headed hhs (in 10%) |  | -0.030 | 0.047 | 0.526 | 0.017 | 0.068 | 0.803 |
| % Extr. low income (in 10%) |  | 0.073 | 0.030 | 0.015^*^ | 0.035 | 0.044 | 0.425 |
| % Hhers. aged 65+ (in 10%) |  | -0.190 | 0.041 | 0.000^***^ | -0.141 | 0.060 | 0.019^*^ |
| Months on waiting list |  | -0.004 | 0.003 | 0.078 | -0.007 | 0.003 | 0.022^*^ |
| PHA size |  |  |  |  |  |  |  |
| 1 - 99 units |  | -1.819 | 0.174 | 0.000^***^ | -1.889 | 0.257 | 0.000^***^ |
| 100 - 299 units |  | -0.642 | 0.162 | 0.000^***^ | -0.765 | 0.228 | 0.001^**^ |
| 300 - 499 units |  |  |  |  |  |  |  |
| 500 - 999 units |  | 0.114 | 0.166 | 0.493 | -0.066 | 0.250 | 0.790 |
| 1000+ units |  | 0.536 | 0.171 | 0.002^**^ | 0.386 | 0.259 | 0.136 |
| *Service Area* |  |  |  |  |  |  |  |
| % Black pop (in 10%) |  | -0.076 | 0.060 | 0.203 | -0.098 | 0.094 | 0.294 |
| % Hispanic pop (in 10%) |  | -0.046 | 0.063 | 0.467 | -0.011 | 0.102 | 0.911 |
| % Female-headed households |  | 0.014 | 0.014 | 0.301 | 0.003 | 0.020 | 0.877 |
| % Families in poverty |  | 0.001 | 0.009 | 0.886 | 0.016 | 0.013 | 0.242 |
| % Unemployed |  | 0.004 | 0.013 | 0.778 | -0.003 | 0.020 | 0.885 |
| % Renting households |  | 0.009 | 0.006 | 0.131 | -0.001 | 0.010 | 0.904 |
| % Households with children |  | 0.010 | 0.009 | 0.294 | -0.001 | 0.016 | 0.950 |
| Property value (in $1000s) |  | 0.001 | 0.001 | 0.189 | 0.000 | 0.001 | 0.825 |
| Eviction filing rate (logged) |  | 0.095 | 0.025 | 0.000^***^ | 0.077 | 0.037 | 0.036^*^ |
| Constant |  | -1.505 | 0.547 | 0.006^**^ | -2.708 | 1.663 | 0.103 |
| R^2^ (overall) |  | 0.560 | | | 0.625 | | |
| N |  | 7,821 (1,243 PHAs) | | | 4,393 (705 PHAs) | | |

Note: Both the PHA filing rate (outcome) and filing rate for private rentals in the surrounding area (covariate) exclude multiple cases filed against the same household. *** p< 0.001; ** p< 0.01; * p< 0.05. Fixed-effects for states (for the full sample) or counties (for the within-county sample) and years were included in the model but not shown in the table due to space considerations.

**Table C3.** Results from longitudinal linear regression model with random effects excluding multiple (“serial”) filings against the same households, 2006-2016

| **Variable** | | **Coeff.** | **SE** | **Sig.** |
| --- | --- | --- | --- | --- |
| *Public Housing Authority* |  | |  |  |
| % Black tenants (in 10%) | 0.091 | | 0.034 | 0.007** |
| % Hispanic tenants (in 10%) | -0.093 | | 0.048 | 0.049* |
| % Households with children (in 10%) | 0.060 | | 0.043 | 0.166 |
| % Female-headed households (in 10%) | -0.026 | | 0.048 | 0.582 |
| % Households extremely low income (in 10%) | 0.062 | | 0.030 | 0.040* |
| % Householders aged 65+ (in 10%) | -0.216 | | 0.041 | 0.000*** |
| Months on waiting list | -0.004 | | 0.003 | 0.099 |
| PHA size |  | |  |  |
| 1 - 99 units | -1.753 | | 0.188 | 0.000*** |
| 100 - 299 units | -0.634 | | 0.177 | 0.000*** |
| 300 - 499 units | (*reference*) | | | |
| 500 - 999 units | 0.150 | | 0.183 | 0.413 |
| 1000+ units | 0.522 | | 0.191 | 0.006** |
| *Service Area* |  | |  |  |
| % Black population (in 10%) | -0.083 | | 0.063 | 0.187 |
| % Hispanic population (in 10%) | -0.032 | | 0.066 | 0.622 |
| % Female-headed households | 0.019 | | 0.014 | 0.178 |
| % Families in poverty | 0.002 | | 0.009 | 0.827 |
| % Unemployed | 0.005 | | 0.014 | 0.730 |
| % Renting households | 0.007 | | 0.006 | 0.282 |
| % Households with children | 0.009 | | 0.009 | 0.322 |
| Property value (in $1000s) | 0.001 | | 0.001 | 0.277 |
| Eviction filing rate (logged) | 0.092 | | 0.024 | 0.000*** |
| Constant | -1.325 | | 0.560 | 0.018* |
| R^2^ = 0.569 (overall) | | | | |
| N = 7,177 PHA-years (1,207 PHAs) | | | | |

Note: *** p< 0.001; ** p< 0.01; * p< 0.05. Fixed-effects for states and years were included in the model but not shown in the table due to space considerations.

**Table C4.** Results from longitudinal linear regression model with random effects, excluding PHA-years with more than 5% of filings identified by address only, 2006-2016

**D. Supplementary Figures**


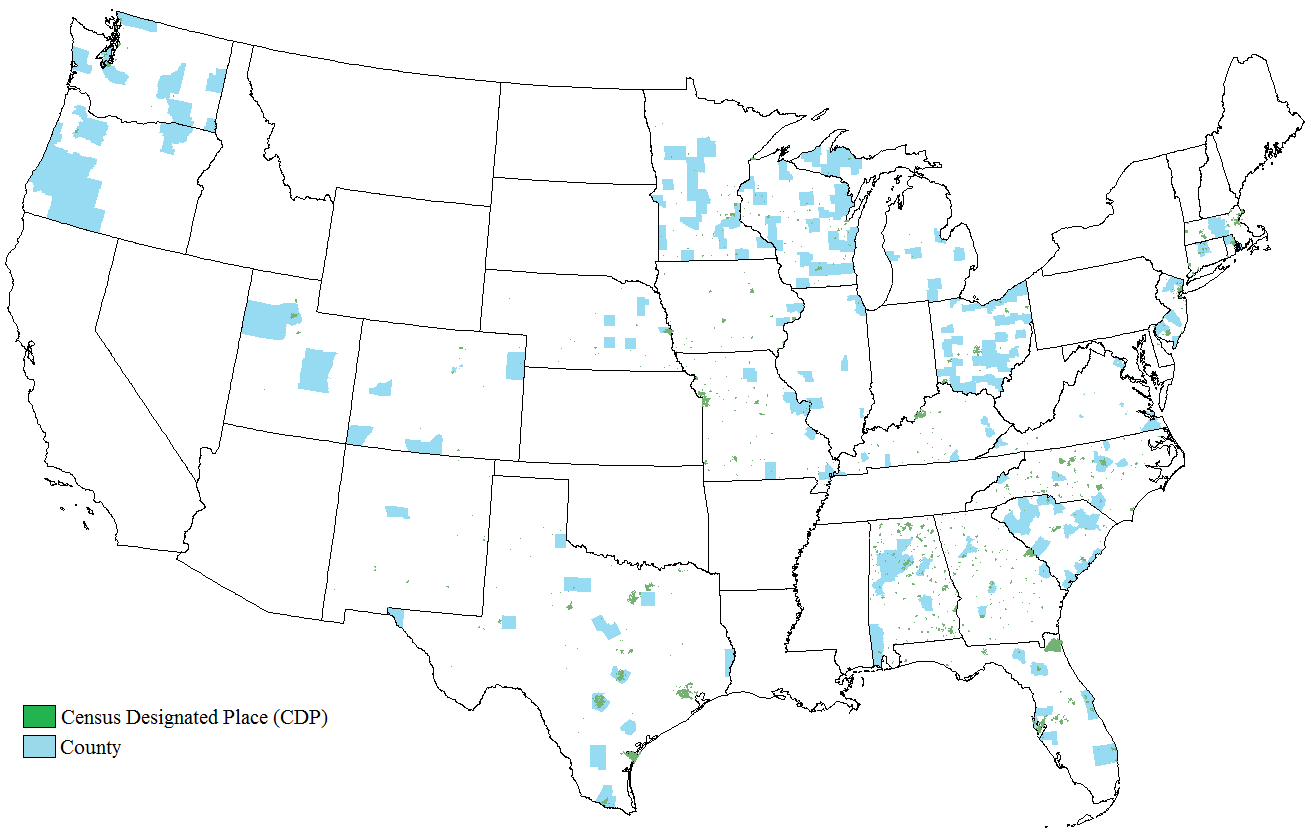


Note: Neither Alaska nor Hawaii had PHAs included in the sample and, therefore, are not shown on the map.

**Figure D1.** Counties and Census Designated Places (CDPs) with PHAs included in sample. N=1,111 CDPs and N=132 counties across 26 states.

| **(A) PHA** | **(B) Private** |
| --- | --- |
| 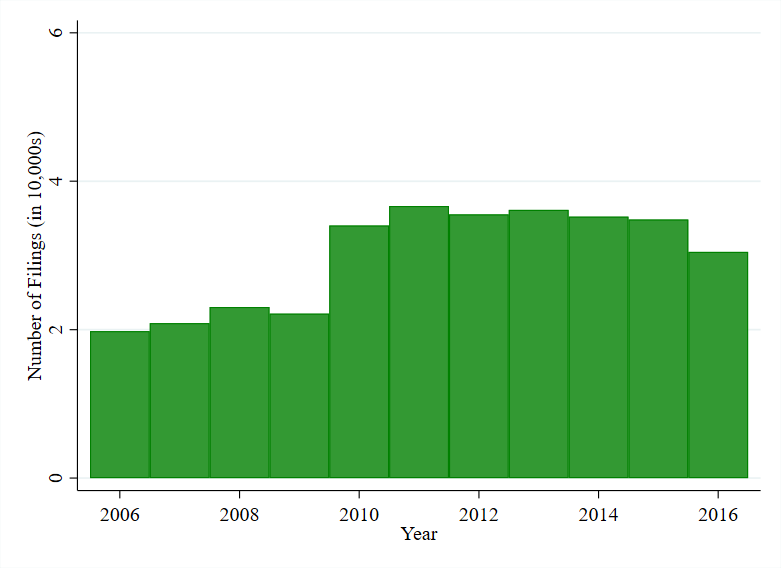 | 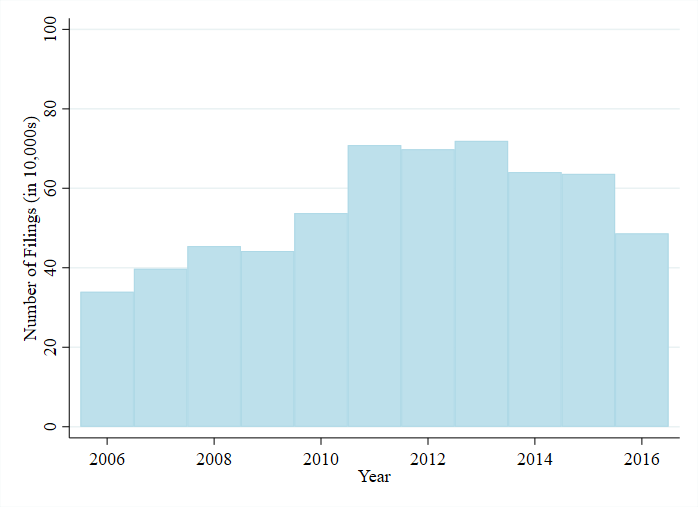 |

**Figure D2.** Annual PHA (A) and private (non-PHA) (B) eviction filings, 2006-2016


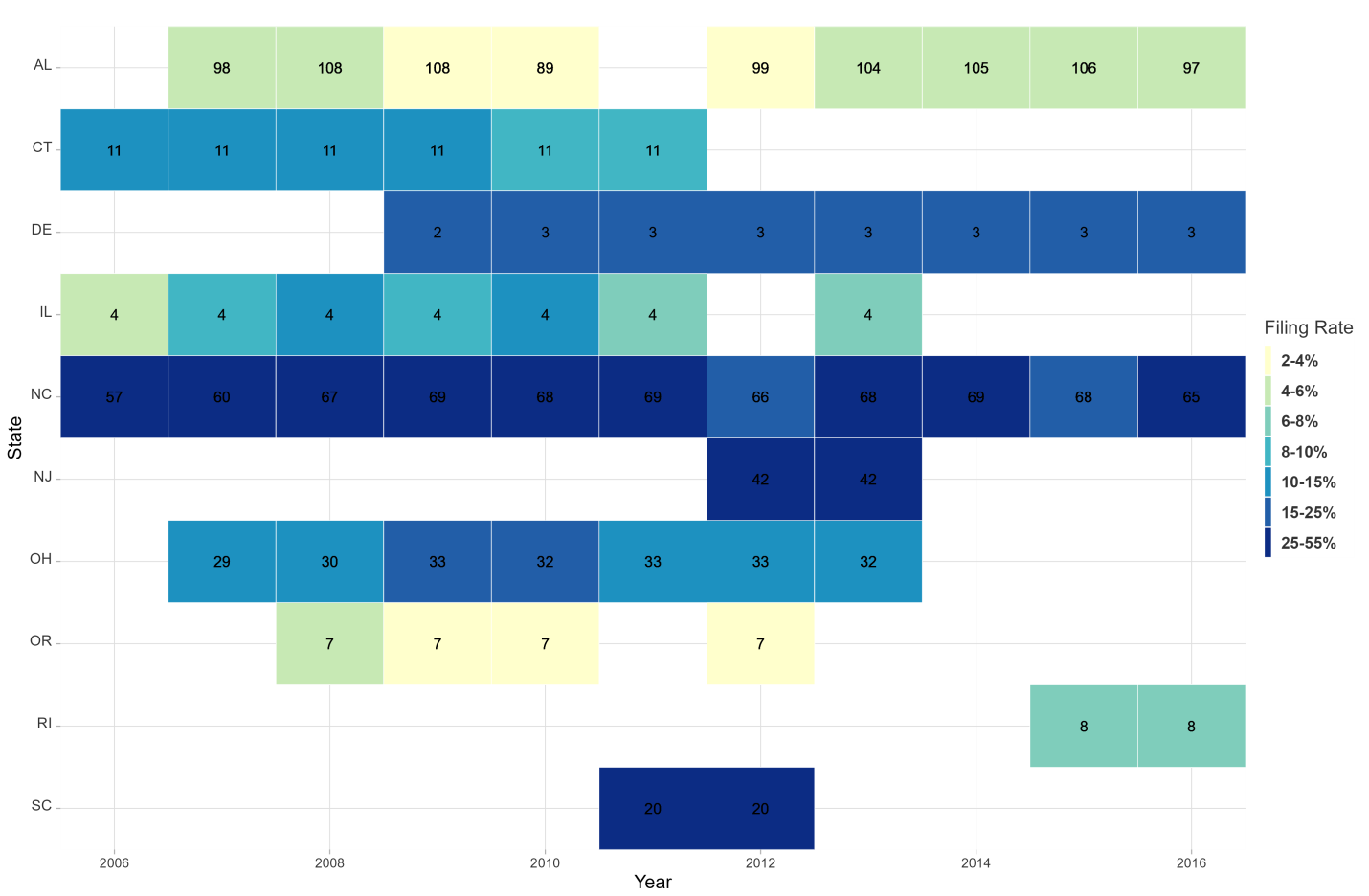


Note: N=2,147. PHAs were considered to be consistently represented if included in at least two-thirds (66.7%) of state years with any verified filing date. State-years were included in the figure if at least two-thirds of sample PHAs were consistently represented and the total number of sample PHAs for that year constituted at least two-thirds of the maximum number of PHAs observed in any year for each state. Numbers within the boxes show the number of PHAs included in the average rate calculation in that state-year. Averages were weighted by number of occupied units in the PHAs.

**Figure D3.** Longitudinal weighted PHA eviction filing rates, by state, 2006-2016


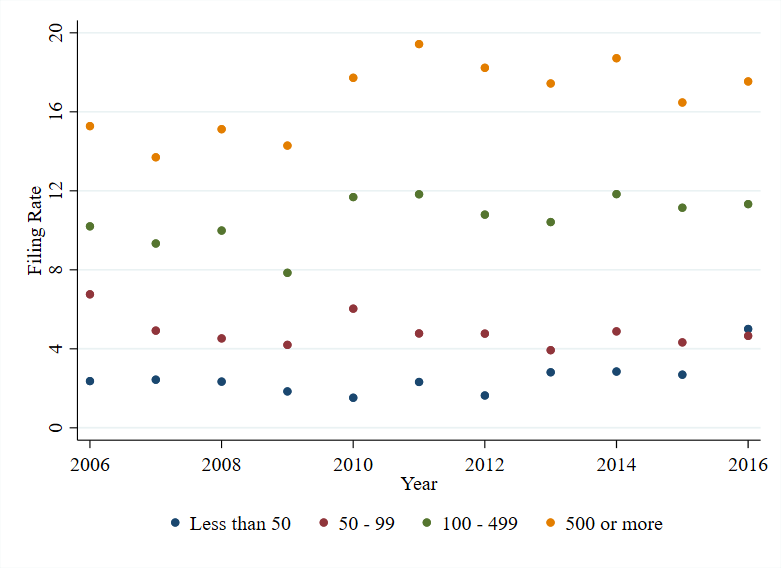


Note: Denominator for filing rate is occupied units.

**Figure D4**. Average PHA eviction filing rates, by number of occupied PHA units, 2006-2016


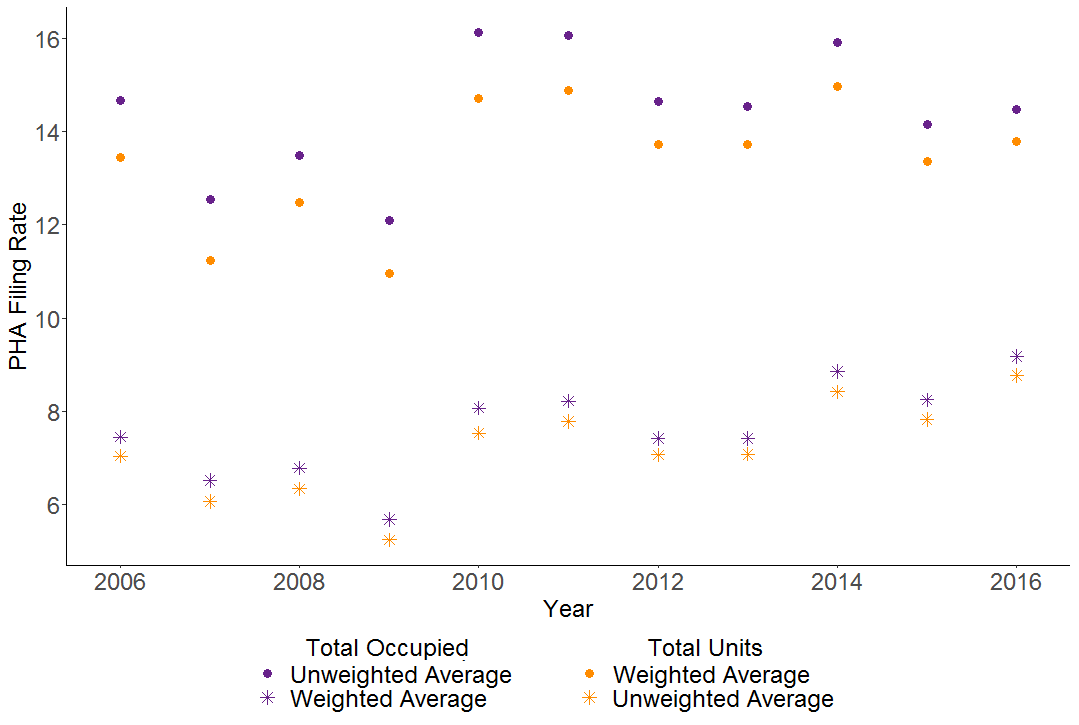


**Figure D5**. Weighted and unweighted PHA eviction filing rates, by occupied and total units, 2006-2016


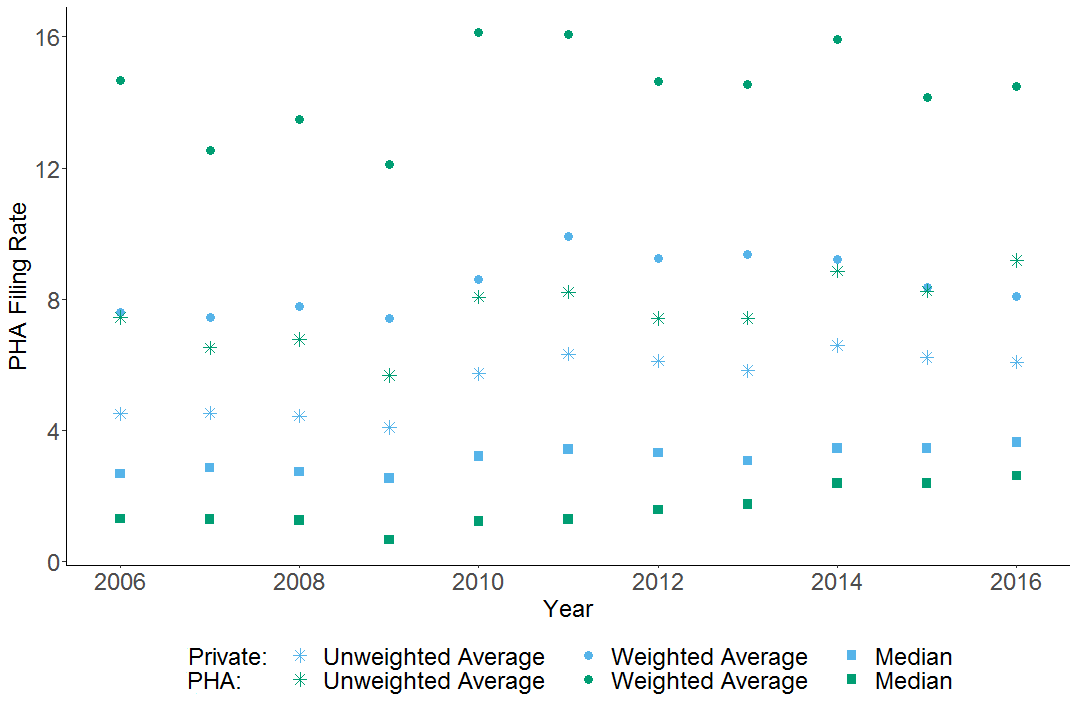


Note: Denominator for filing rate is occupied units.

**Figure D6**. Average and median eviction filing rates, by housing type, 2006-2016


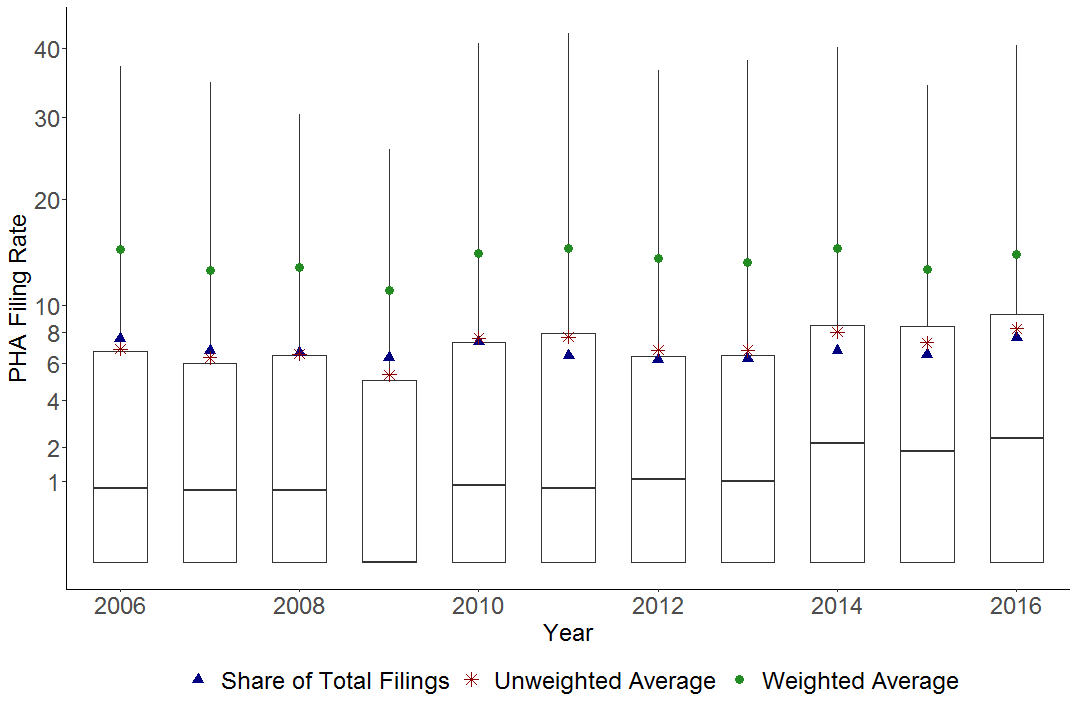


Note: Box plots show 5^th^ through 95^th^ percentiles of PHA filing rates annually. Lower bound of box marks 25^th^ percentile, middle line the median (50^th^ percentile), and upper bound of box the 75^th^ percentile. Summary statistics—share of total eviction filings attributable to PHAs, unweighted mean PHA filing rate, and weighted PHA mean filing rate—shown by points. Y-axis plotted on square root scale due to right skew present in distribution of filing rates.

**Figure D7.** Distribution of PHA filing rates with summary statistics for share of total filings, unweighted and weighted average filing rates, excluding PHA-years with more than 5% of filings identified by address only, 2006-2016.

1. Although it was difficult to adjudicate between distinct PHAs programmatically due to name similarities, we were able to use these matches to identify a few additional public housing cases that were not captured in Section 1a. We marked cases as public housing when the reclink similarity score between the PHA and plaintiff names was at least 0.85 and the plaintiff name contained some variation of “housing.” [↑](#footnote-ref-1)
2. This category was created from an open-ended question asking whether there were any other specific groups that were given preference for placement in public housing units. Some form of work requirement was one of the most frequent responses, which may have prompted HUD to create a specific indicator for this group when tabulating survey results. [↑](#footnote-ref-2)
3. These missing values were not explicitly coded indicators of non-response. Other survey questions included values for “clear my response” or “refused,” but the questions concerning specific waitlist preferences were coded only as “Selected” or “Not Selected” with no additional explanation for missing values. [↑](#footnote-ref-3)
